# Pharmacological inhibition of MutT homolog 1 (MTH1) in allergic airway inflammation as a novel treatment strategy

**DOI:** 10.1186/s12931-025-03175-z

**Published:** 2025-03-14

**Authors:** Anna Adler, Jesper Bergwik, Médea Padra, Praveen Papareddy, Tobias Schmidt, Madelene Dahlgren, Robin Kahn, Ulrika Warpman Berglund, Arne Egesten

**Affiliations:** 1 Division of Respiratory Medicine, Allergology, & Palliative Medicine, Department of Clinical Sciences Lund and Skåne University Hospital, Lund, Sweden; 2 Department of Laboratory Medicine, Lund University, Lund, Sweden; 3 Division of Pediatrics Department of Clinical Sciences Lund, Lund University and Skåne University Hospital, Lund, Sweden; 4 Wallenberg Center for Molecular Medicine, Lund, Sweden; 5 Lung Biology, Department of Experimental Medical Sciences, Lund University, Lund, Sweden; 6 Science for Life Laboratory, Department of Oncology-Pathology, Karolinska Institutet, Solna, Sweden; 7 Oxcia AB, Stockholm, Sweden

**Keywords:** Asthma, MTH1, TH1579, T cells, Type 2 inflammation

## Abstract

**Background:**

Despite progress in the treatment of asthma, there is an unmet need for additional therapeutic strategies, not least to avoid side-effects of corticosteroids. The enzyme MutT homolog 1 (MTH1) hydrolyzes oxidized purines and prevents their insertion to DNA. Small molecule inhibition of MTH1 has shown promising therapeutic effects in both cancer and inflammatory conditions. In this study, a small molecule inhibitor of MTH1 (TH1579), was investigated in models of allergic inflammation.

**Methods:**

In vitro, effects on T cell proliferation and apoptosis were investigated. Furthermore, a murine model, using female BALB/c mice, of OVA-induced allergic airway inflammation was used to investigate effects from MTH1-inhibition in vivo.

**Results:**

Inhibition of MTH1 prevented T cell proliferation in vitro and induced apoptosis in isolated human CD4^+^ T cells. However, the viability of isolated human eosinophils was unaffected by MTH1 inhibition in vitro. Pharmacological inhibition of MTH1 in a murine model of allergic airway inflammation reduced mucus production, recruitment of inflammatory cells, such as T cells and eosinophils in the BAL fluid and lung tissue, reduced plasma levels of total IgE and OVA-specific IgE, IgG, and IgG1, as well as reduced IL-13 levels in BAL fluid, lung tissue and plasma.

**Conclusion:**

MTH1 inhibition reduced proliferation and promoted apoptosis of T cells in vitro. In vivo, TH1579 dampened the type 2 associated immune response in a murine model. These findings suggest that MTH1 could serve as a novel target to treat allergic airway inflammation.

**Supplementary Information:**

The online version contains supplementary material available at 10.1186/s12931-025-03175-z.

## Introduction

Asthma is one of the most common chronic inflammatory airway diseases affecting around 300 million individuals worldwide [1, 2]. Around 60–80% of adult asthma cases are associated with a longstanding and excessive type 2 immune response against airborne allergens [3, 4]. Macrophages and dendritic cells present allergens to naïve CD4^+^ T helper cells that proliferate, increase their intracellular reactive oxygen species (ROS) levels, being skewed to differentiate toward a T helper 2 (Th2) phenotype. In addition, they secrete the prototypic type 2 cytokines IL-4, IL-5 and IL-13 [5–7]. Subsequently, plasma cells produce allergen-specific IgE that, when receptor-bound and crosslinked on the surface of mast cells and basophils, triggers release of pro-inflammatory mediators. These promote inflammation, bronchoconstriction, edema, mucus production, and cause tissue damage in the lung. Inflammation in asthma can lead to irreversible airway remodeling due to subepithelial fibrosis, extracellular matrix degradation, an increase in smooth muscle cell mass, and goblet cell hyperplasia. The presence of eosinophils is a hallmark of type 2 inflammation. IL-5 promotes eosinophil differentiation in the bone marrow, stimulates their recruitment and activation at the sites of inflammation, as well as delaying their apoptosis. Furthermore, activated eosinophils release pro-inflammatory mediators that amplify inflammation and tissue damage in the lungs [3, 4].

Current treatments of asthma rely mainly on β_2_-adrenergic receptor agonists, acting as bronchodilators, and inhaled corticosteroids, reducing inflammation. In addition, muscarinic antagonists, leukotriene receptor antagonists, and oral corticosteroids can be added to further reduce inflammation and alleviate symptoms [2]. In recent years the design of several new targeted biological therapies, including monoclonal antibodies against key mediators of type 2 inflammation such as IgE, IL-4, IL-5 and IL-13, has been inspired by the increased understanding of the type 2 inflammatory network [3]. However, there is still a need for new pharmacological approaches to modulate the dysregulated airway inflammation in allergic asthma, in particular to reduce the need of corticosteroids that have severe side-effects (osteoporosis, metabolic disturbances including diabetes, and mood disorders).

**Fig. 1:**
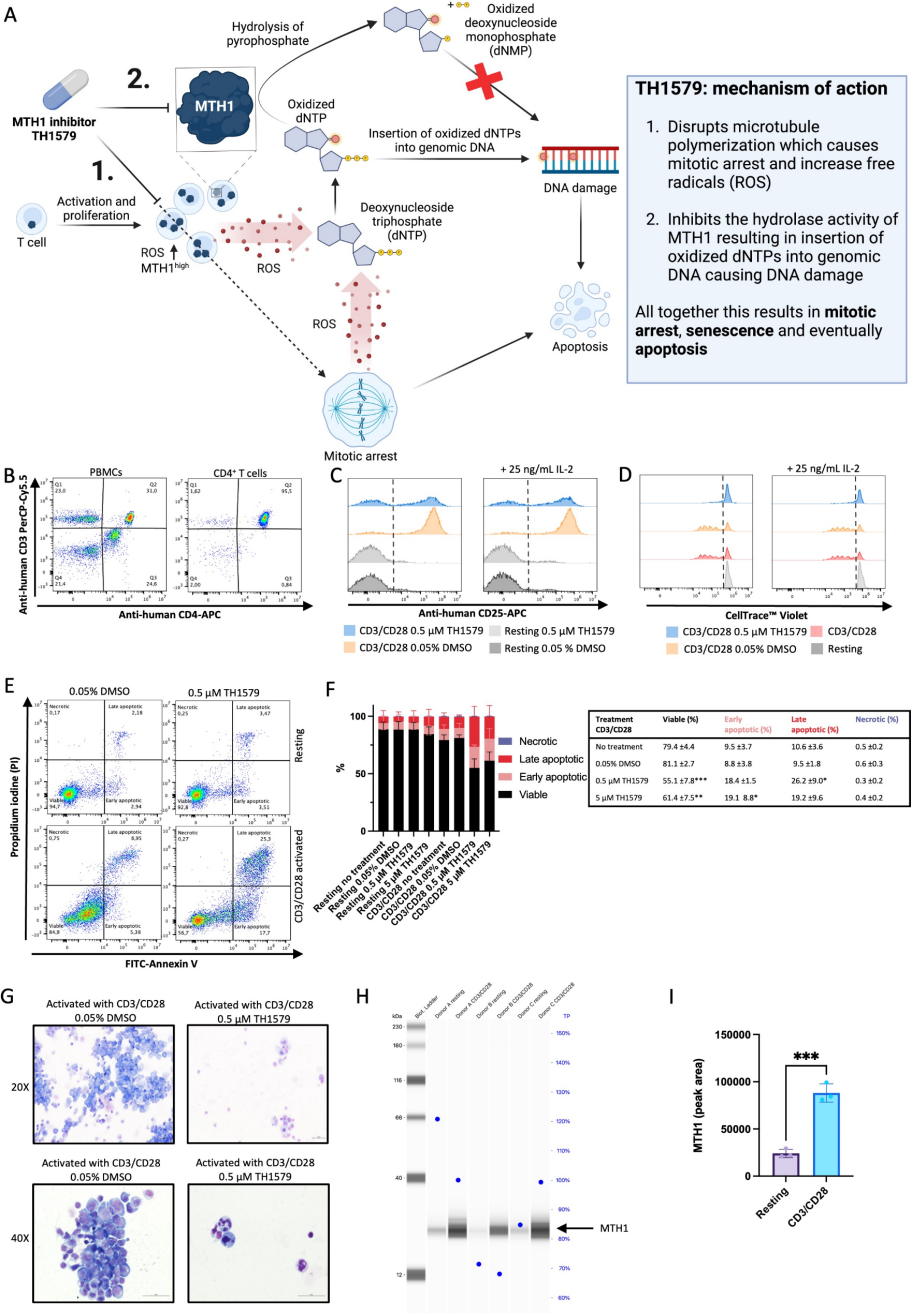
**A–G**. (**A**) Schematic overview of the enzymatic activity of MTH1 and the proposed mechanism of action of the MTH1 inhibitor TH1579. (**B**) Representative flow cytometric analysis of expression of CD3 and CD4 in human PMBCs and isolated CD4^+^ T cells, pre- and post-isolation (*n* = 10). (**C**) Representative histograms of CD25 surface marker staining on CD4^+^ T cells after 96 h ± CD3/CD28 stimulation (*n* = 4). (**D**) Cell proliferation of activated (CD3/CD28) human CD4^+^ T cells, in the abscense or presence of TH1579, was measured by intracellular fluorescent dye dilution (*n* = 4). (**E**) Representative flow cytometry dot plots of double stained Annexin V-FITC/Propidium iodine (PI) CD4^+^ T cells for control cells (vehicle), and cells treated with TH1579 (0.5 µM; *n* = 4). (**F**) Summary of CD4^+^ T cell apoptosis data (*n* = 4). (**G**) Representative images of May Grünwald-Giemsa stained cytospins (scale bar = 5 µm) of human CD4^+^ T cells after 96 h after CD3/CD28 stimulation in the abscense or presence of TH1579. (**H**) Virtual blot; quantification of MTH1 in human CD4^+^ T cells with or without CD3/CD28 stimulation for 96 h under reduced conditions using Jess automated capillary western blot. Total protein (TP) levels in each sample are presented in % as blue dots. (**I**) Total peak area of MTH1 detected in Jess, at 25 kDa ± 10%, after total protein normalization (*n* = 3). The results are displayed as mean ± SD. Statistical comparisons with tree or more groups were performed using one-way ANOVA with Dunnett’s post hoc test comparing the mean of each group to the group treated with 0.05% DMSO. Two-tailed unpaired *t*-test was used to compare two groups. (****P < 0.0001, ***P < 0.001, **P < 0.01, *P < 0.05)

Recent studies have proposed inhibition of human nudix hydrolase MutT Homolog 1 (MTH1/ NUDT1) as a potential treatment strategy in T cell driven diseases [8, 9]. MTH1 is a 18-22.5 kDa enzyme that sanitizes the cellular deoxynucleotide triphosphate (dNTP) pool by catalyzing the hydrolysis of oxidized purine nucleoside triphosphates, dNTPs, to monophosphates (dNMPs). Cancer cells, as well as activated proliferating T cells, have an altered redox balance and upregulate MTH1 to avoid insertion of oxidized dNTPs into their DNA (Fig. 1A), making MTH1^high^ cells more sensitive to MTH1 inhibition [10, 11]. MTH1 inhibition introduces additional oxidative stress in MTH^high^ cells and ensures that the cells cannot prevent oxidized DNA damage. Recent studies, using the small molecule MTH1 inhibitor TH1579 (also known under the names OXC-101 and Karonudib), have shown selective suppression of activated T cells. This resulted in increased presence of the modified histone γH2AX foci, reflecting DNA strandbreaks and apoptosis [9, 12, 13]. TH1579 has a dual mechanism where it disrupts microtubule polymerization in proliferating cells, which causes mitotic arrest and increases production of reactive oxygen species (ROS). Importantly, it also inhibits the hydrolase activity of MTH1 resulting in insertion of oxidized dNTPs into genomic DNA. All together this results in mitotic arrest, senescence and eventually apoptosis (Fig. 1A) [9, 11]. Since increased production of ROS, followed by upregulation of MTH1, is seen in proliferating T cells, which orchestrate allergic inflammation, MTH1 may provide a novel therapeutic target in allergic asthma [9, 14]. The aim of this study was to evaluate the treatment potential of the MTH1 inhibitor TH1579 in allergic airway inflammation to specifically target activated T cells using both in vitro and in vivo models.

## Materials and methods

### Ethical approval

The use of human blood from apparently healthy volunteers was approved by the local ethical review board (no. 2015/801) and performed in accordance with the amended Declaration of Helsinki and after informed consent. All mouse experiments were approved by the Malmö-Lund Animal Care Ethics Committee (ethical permits no. M3802-19 and no. 18-20687/2023).

### In vitro experiments – human CD4+ T cells and eosinophils

Detailed information is provided in Supplementary Materials and Methods.

### Animals

Female BALB/c mice were purchased from Janvier (Le Genest-Saint-Isle, France) and maintained for at least two weeks prior to initiation of experiments. Housing conditions and experimental handling were performed according to the guidelines for animal experimentation and welfare at Lund University. The mice were housed in plastic cages with absorbent bedding material and in a controlled environment (temperature, light/dark cycle, food, and water *ad libitum*). The mice were nine weeks old at the start of the experiment.

### In vivo experiments

Allergic airway inflammation was induced in BALB/c mice by sensitization with 20 µg of ovalbumin (OVA, cat#vac-pova; EndoFit™, InvivoGen, Toulouse, France) administrated by intraperitoneal injection (i.p.) in 150 µL alum (1:10) on day 0 and 7. On day 14, 16, 18, and 20 the mice were challenged with OVA using intranasal (i.n.) administration of 50 µg OVA (50 µL of 1 mg/mL OVA) dissolved in sterile endotoxin-free water. PBS was used as a negative control. An i.p. injection of either TH1579 (60 mg/kg, Oxcia, Stockholm, Sweden) dissolved in 20% hydroxypropyl-β-cyclodextrins (HPβCD; cat#332607; Sigma-Aldrich, Saint Louis, MI) in acetate buffer (pH 4.5), dexamethasone (2 mg/kg; cat#D4902; Sigma-Aldrich) dissolved in HPβCD (20%), or vehicle (20% of HPβCD) was administered one hour before each OVA challenge. The mice were randomly allocated into five groups: treated with vehicle and OVA challenged (VO, *n* = 5; initially six mice but one mouse had to be euthanized during the OVA sensitization), treated with TH1579 and OVA challenged (TO, *n* = 6; *n* = 5 for BAL fluid samples used in flow cytometric analysis due to technical issues), treated with dexamethasone and OVA challenged (DO, *n* = 6), treated with TH1579 and PBS challenged (TP, *n* = 4), and treated with vehicle and PBS challenged (VP, *n* = 3) (see Table S1). The mice were sacrificed on day 21 and lungs, spleens, plasma and bronchoalveolar lavage (BAL) fluid were collected, and analyzed for plasma antibody levels, inflammatory cell infiltration using flow cytometry (see antibody panel in Table S2), lung histology, mucus production, cytokine levels, and mRNA expression of 84 genes in the murine lung related to allergy and asthma, as well as the mRNA expression of *Nudt1*. Detailed information of all the methods can be found in the online supplement.

### Statistical analysis

Analysis of differences between three or more groups was calculated using one-way ANOVA with Dunnett’s post hoc test, comparing the mean of each group with VO. Statistical testing was performed using GraphPad Prism 10, version 10.1.1 (GraphPad Software, San Diego, CA), and the statistical significance was defined as ****P < 0.0001, ***P < 0.001, **P < 0.01, *P < 0.05. The results are displayed as mean ±SD.

## Results

### Activated CD4+ T cells are sensitive to MTH1 inhibition in vitro

Human CD4^+^ T cells were isolated from human whole blood (*n* = 10 donors) with a purity of > 90% (CD3^high^ and CD4^high^), as assessed by flow cytometry (Fig. 1B). The isolated CD4^+^ T cells were subsequently activated with an antibody cocktail consisting of CD3/CD28 for 96 h. Upregulation of CD25 was used to confirm cell activation (Fig. 1C and S1). Treatment with TH1579 reduced the number of CD25^high^ cells, indicating less activation. Flow cytometric analysis of activated CD4^+^ T cells stained with a fluorescent dye (CellTrace™ Violet), to track cell proliferation by fluorescent dye dilution, revealed that TH1579 inhibited T cell proliferation (Fig. 1D). TH1579 induced apoptosis in activated CD4^+^ T cells whereas resting CD4^+^ T cells were not affected (Fig. 1E and F; gating strategies and statistical analysis are shown in Fig. S2 and Table S3). May-Grünwald Giemsa was used to stain cytospins to quantify cell numbers and morphology of resting and activated CD4^+^ T cells that had been incubated in the absence or presence of TH1579. This further revealed the sensitivity of MTH1 inhibition in activated CD4^+^ T cells, as the number of TH1579-treated cells were reduced compared to controls. Many cells appeared apoptotic (membrane blebbing, rounded nuclei, and homogenously dispersed chromatin), as well as showed signs of mitotic arrest (Fig. 1G). Upregulation of MTH1 in activated T cells was demonstrated using Jess, an automated capillary western blot (Fig. 1H and I). Since the presence of eosinophils is a hallmark of allergic inflammation and these cells are end-stage effector cells having a capability of high ROS production, possible effect of MTH1 inhibition was investigated. However, TH1579 did not promote apoptosis in isolated human eosinophils (Figs. S3A–J).

### MTH1 inhibition decreases plasma levels of OVA-specific IgE, IgG and IgG1 in vivo

**Fig. 2:**
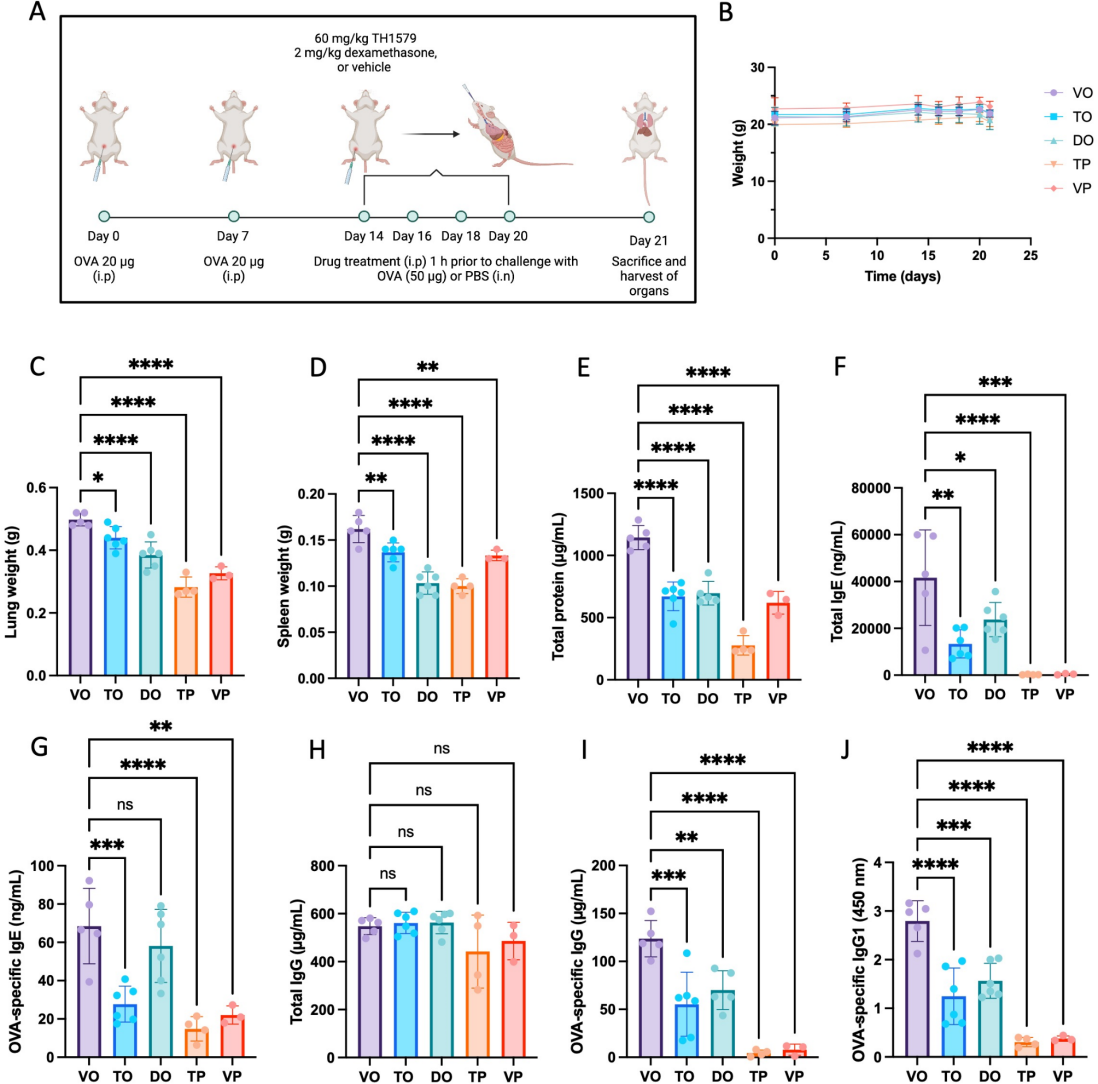
**A–J**. (**A**) Overview of the experimental design of the female BALB/c mice model of ovalbumin (OVA)-induced allergic airway inflammation. (**B**) Body weights remained stable in all groups over the 21 days. (**C**) Lung and (**D**) spleen weight comparison between the groups showing a significant weight increase in the lungs and spleens of the Vehicle/OVA (VO, *n* = 5), group compared to the TH1579/OVA (TO, *n* = 6), dexamethasone/OVA (DO, *n* = 6), TH1579/PBS (TP, *n* = 4), and vehicle/PBS (VP, *n* = 3) groups. (**E**) Total protein concentration in the bronchoalveolar lavage (BAL) fluid. Lower protein levels were observed in the TH1579 and dexamethasone treated groups compared to mice only receiving vehicle. Quantification of the following antibodies in plasma using ELISA specific for; (**F**) total IgE, (**G**) OVA-specific IgE, (**H**) total IgG, (**I**) OVA-specific IgG, and (**J**) OVA-specific IgG1. Mice treated with the MTH1-inhibitor TH1579 had lower levels of total IgE, OVA-specific IgE, OVA-specific IgG and OVA-specific IgG1 compared to the VO-group. The results are displayed as mean ± SD. Statistical comparisons were performed using one-way ANOVA with Dunnett’s post hoc test (****P < 0.0001, ***P < 0.001, **P < 0.01, *P < 0.05)

Allergic airway inflammation was induced in mice by OVA-sensitization and challenge, and an i.p. administration of TH1579 or vehicle was performed 1 h prior to each challenge (Fig. 2A). The administration schedule and dose of TH1579 was based on preliminary experiments. Mice treated with a lower dose of TH1579 (45 mg/kg) showed trends of reduced airway inflammation but no significant difference when compared to vehicle/OVA (S4-6). Whereas mice treated with a higher dose, i.e. TH1579 (90 mg/kg), showed signs of discomfort and two out of five mice were euthanized. Therefore, a dose of 60 mg/kg TH1579 was chosen for subsequent experiments. A dose of 2 mg/kg dexamethasone was chosen as it has been shown to alleviate allergic airway inflammation in a previous study using a murine OVA-model [15]. However, in this study we cannot exclude that a higher dose of dexamethasone may reduce the inflammation further [16]. No differences in weight change were observed between the OVA-challenged groups (Fig. 2B). TH1579 treatment reduced the weight of the lungs and spleens in the TH1579/OVA-group compared to the vehicle/OVA-group (Fig. 2C and D). Total protein concentration in BAL fluid was quantified using a BCA assay, showing less protein in BAL fluid of the TH1579/OVA-group compared to the vehicle/OVA-group (Fig. 2E). Increased lung and spleen weights, as well as protein-levels of the BAL fluid were observed, suggesting tissue inflammation and capillary leakage. Plasma-levels of total IgE, OVA-specific IgE, OVA-specific IgG and OVA-specific IgG1 were all significantly lower in the TH1579/OVA-group compared to vehicle/OVA, whereas the levels of total IgG were similar in all groups (Fig. 2F-J).

### MTH1 inhibition reduces allergic airway inflammation in vivo

**Fig. 3:**
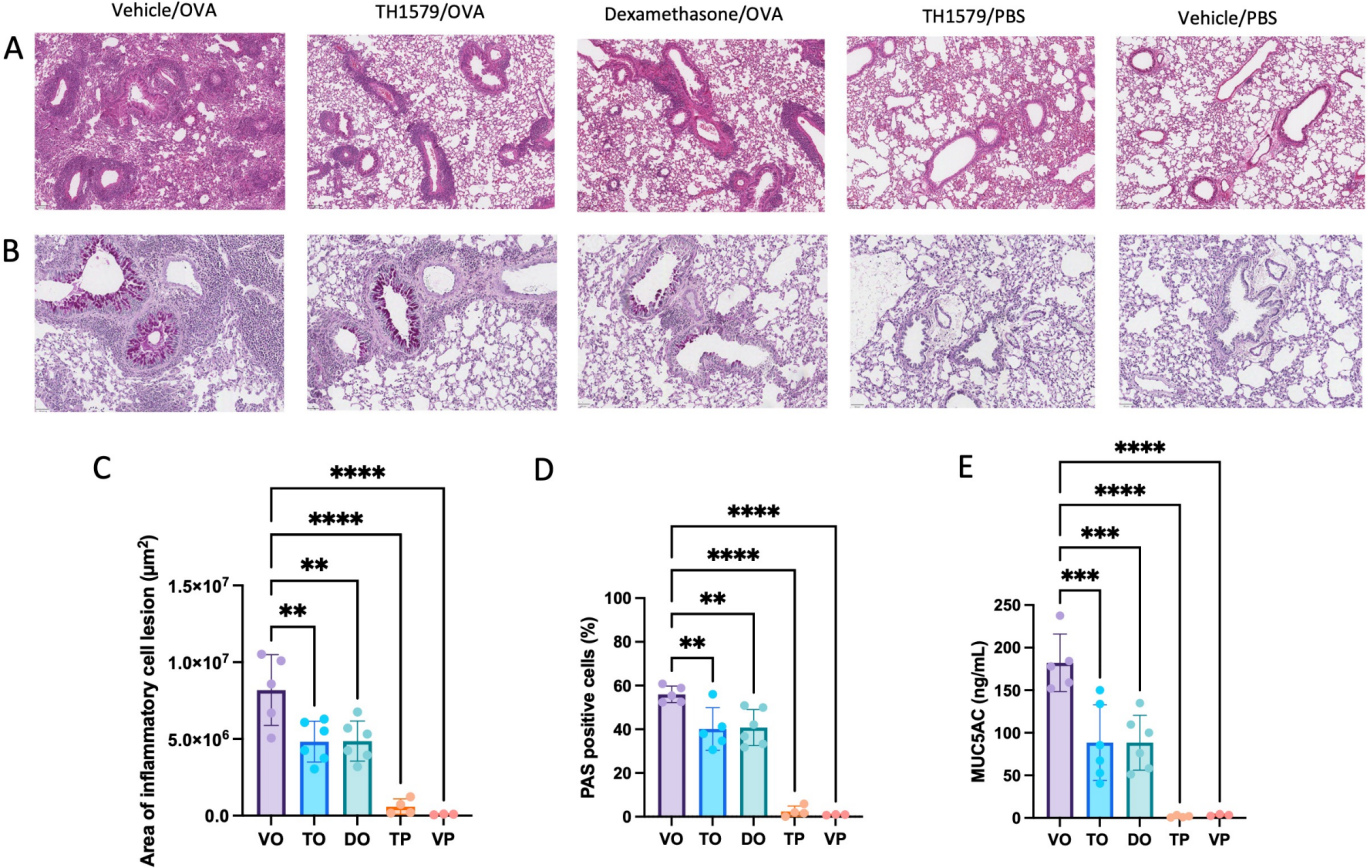
**A–E**. Haemotoxylin and eosin (H&E)- and periodic acid Schiff (PAS)-staining of murine lung tissue sections from OVA-challenged mice with and without TH1579 treatment. (**A**) Representative images of H&E-stained lungs. Scale bar: 100 μm, 30x magnification. (**B**) Representative images of PAS-stained lungs. Mucus and mucus containing cells (mainly goblet cells) stain positive for PAS (magenta). Scale bar: 50 μm, 40x magnification. (**C**) Areas of inflammatory cell infiltrates in H&E stained lung sections were quantified using computer-assisted morphometrical analysis (QuPath). The measured inflammatory area values were normalized to the whole area of each tissue section. (**D**) Mucus production was analyzed by calculating PAS positive cells (%) in the airways using computer-assisted morphometrical analysis (QuPath). The average percentage of PAS positive cells in four airways per tissue section was measured. (**E**) Quantification of MUC5AC levels in the bronchoalveolar lavage (BAL) fluid using ELISA. Vehicle/OVA (VO, *n* = 5), TH1579/OVA (TO, *n* = 6), dexamethasone/OVA (DO, *n* = 6), TH1579/PBS (TP, *n* = 4), and vehicle/PBS (VP, *n* = 3). The results are displayed as mean ±SD. Statistical comparisons were performed using one-way ANOVA with Dunnett’s post hoc test (****P < 0.0001, ***P < 0.001, **P < 0.01, *P < 0.05)

Histological analyses, using H&E- and periodic acid-Schiff (PAS)-staining, of lungs collected from the OVA-sensitized and challenged mice were performed (Fig. 3A and B). The control groups, TH1579/PBS and vehicle/PBS showed a similar, normal architecture. In contrast, the vehicle/OVA-group showed increased inflammatory cell infiltration and irregular distribution of air spaces (Fig. 3A and C). Treatment with TH1579 markedly reduced inflammatory lesions in mice with OVA-induced inflammation, which also was the case for mice treated with dexamethasone. Images of the whole H&E- and PAS-stained lungs from all mice can be found in the supplement (Figs. S7A-F and S8A-F). To evaluate a possible goblet cell hyperplasia and increase in mucus production, lung sections were stained with PAS. All OVA-challenged mice displayed signs of increased mucus production and goblet cell hyperplasia. However, compared to vehicle/OVA, the TH1579-treated and OVA-challenged mice displayed a significantly lower number of PAS positive cells (Fig. 3B and D). MUC5AC ELISA was performed to evaluate the levels of MUC5AC in BAL fluid, showing decreased levels of MUC5AC in TH1579/OVA compared with the vehicle/OVA group, supporting the histological observations (Fig. 3E).

### Analysis of cells in BAL fluid and lung tissue

**Fig. 4:**
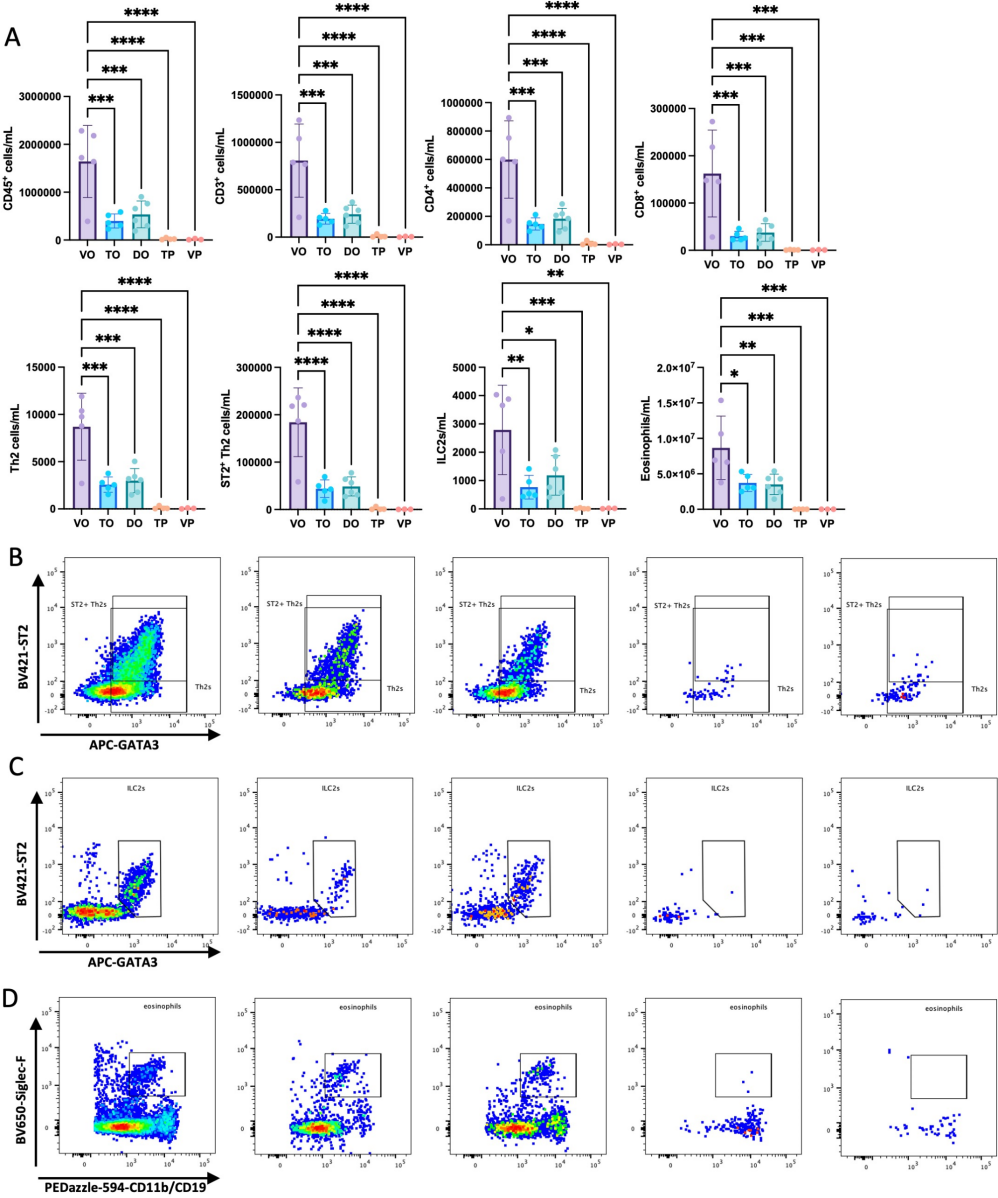
**A–D**. (**A**) Quantification of inflammatory cells in bronchoalveolar lavage (BAL) fluid. Vehicle/OVA (VO, *n* = 5), TH1579/OVA (TO, *n* = 5), dexamethasone/OVA (DO, *n* = 6), TH1579/PBS (TP, *n* = 4), and vehicle/PBS (VP, *n* = 3). Representative dot plots of; (**B**) type 2 helper (Th2) cells (Live/Dead Aqua^–^CD45^+^ CD3^+^CD4^+^GATA3^+^) and ST2^+^Th2 cells (Live/Dead Aqua^–^CD45^+^CD3^+^CD4^+^GATA3^+^ST2^+^), (**C**) type 2 innate lymphoid cells (Live/Dead Aqua^–^CD45^+^CD3^–^GATA3^+^ST2^+^), and (**D**) eosinophils (Live/Dead Aqua^–^CD45^+^CD11b^+^Siglec-F^+^). The results are displayed as mean ± SD. Statistical comparisons were performed using one-way ANOVA with Dunnett’s post hoc test (****P < 0.0001, ***P < 0.001, **P < 0.01, *P < 0.05)

Flow cytometric analysis of BAL fluid revealed reduced numbers of lymphocytes in the TH1579/OVA-group compared to vehicle/OVA. This included T helper (CD4^+^) and cytotoxic (CD8^+^) T cells, Th2 cells, ST2^+^-Th2-cells, type 2 innate lymphoid cells (ILC2), as well as reduced numbers of eosinophils (Fig. 4A-D). Significantly lower numbers of CD4^+^ and CD8^+^ T cells were also observed in lung homogenate of the TH1579/OVA-group compared to vehicle/OVA (S9A). While no significant difference was seen in the numbers of Th2 cells and eosinophils in the lung homogenates, there was a trend towards lower numbers in the group that were exposed to TH1579/OVA in comparison to the group only receiving vehicle/OVA(S9B-D). This shows that administration of TH1579 reduces the number of T cells and recruitment of eosinophils to the lung. Gating strategies are shown in Figs. S10A and B.

### Pharmacological inhibition of MTH1 reduces type 2 associated cytokine levels in BAL fluid, lung and plasma

**Fig. 5:**
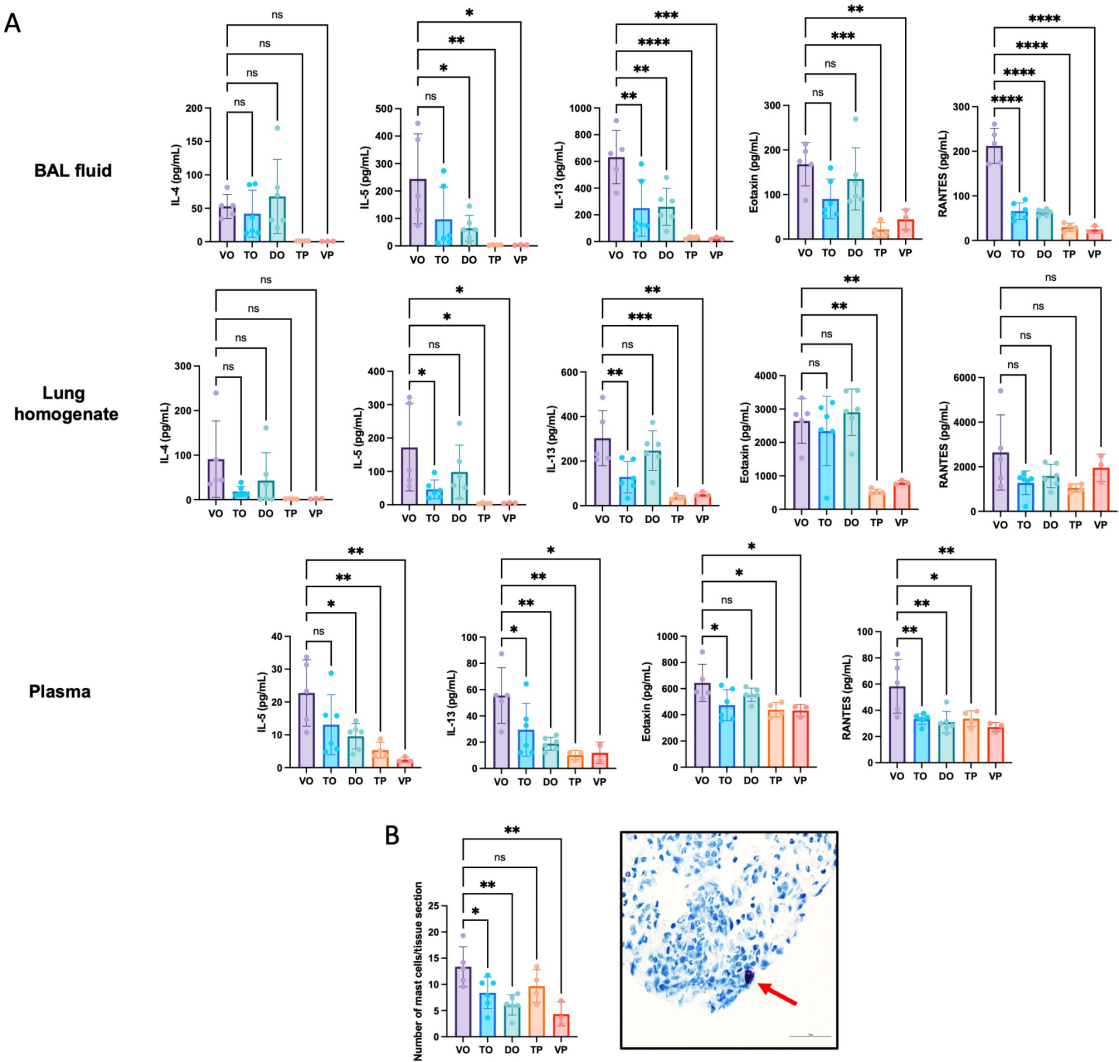
**A–B**. (**A**) Quantification of type 2 associated cytokines, IL-5 and IL-13, eotaxin and RANTES in bronchoalveolar lavage (BAL) fluid, lung tissue homogenate and plasma. (**B**) Mast cell quantification in lung tissue, and representative images of mast cells (red arrow) stained with toluidine blue (40x magnification, scale bar = 5 μm). All mast cells in each section were counted and normalized to the whole area of each tissue section. The results are displayed as mean ±SD. Statistical comparisons were performed using one-way ANOVA with Dunnett’s post hoc test (****P < 0.0001, ***P < 0.001, **P < 0.01, *P < 0.05)

A 23-cytokine multiplex assay was used to measure the levels of pro-inflammatory cytokines in BAL fluid, lung homogenate and plasma collected from mice with OVA-induced allergic airway inflammation. In the vehicle/OVA group, several pro-inflammatory cytokines were increased in BAL fluid, lung homogenate and plasma. Lower levels of several type 2 associated cytokines, such as IL-5 and IL-13, were observed in the TH1579/OVA group (Fig. 5A), supporting reduced inflammation and cell recruitment, including mast cells (Fig. 5B), to the lung by MTH1 inhibition. Cytokine levels in plasma are presented in Fig. S9, with the exception of IL-1α, IL3, IL-4, IL-6, IL12 (p70), G-CSF, GM-CSF and MIP-1β, as the levels were below the detection limits of the assay. The measured concentration of all cytokines analyzed in the BAL fluid, lung homogenate and plasma are included in the supplement (Figs. S11-13).

### OVA-induced inflammation upregulates the expression of *Nudt1* in lung tissue

The expression of 84 genes related to murine allergic inflammation was quantified using a PCR array (Fig. S17A-D). Differences in differential regulation of mRNA were seen when comparing the vehicle/OVA and TH1579/OVA groups (Fig. S17A). Increases in fold regulation were seen in the cases of *Ccl17* (3.4), *Il13ra1* (2.0), *Il9* (2.2) and *Ms4a2* (2.0), while *Il3* (–2.3) was decreased in the mice exposed to TH1579/OVA in comparison to vehicle/OVA. Comparison between the negative control group, vehicle/PBS, and the positive control group, vehicle/OVA, revealed up-regulation of several genes in the vehicle/OVA group (Fig. S17B). No difference was noted in the comparison between the PBS/vehicle and TH1579/PBS, indicating that TH1579 itself does not affect gene expression of the genes analyzed in this panel (Fig. S17C). Differences in fold regulation between vehicle/OVA and the comparative drug, dexamethasone are shown in Fig. S17D. Lastly, expression of *Nudt1*, the gene encoding MTH1, was analyzed in lungs from mice with OVA-induced inflammation and controls respectively. A non-significant trend towards higher expression was seen in mice with OVA-induced inflammation (Fig. S17E). The increase in *Nudt1* expression was similar to that previously reported in human psoriatic skin [8]. The upregulation indicates that MTH1 has important roles in the context of allergic inflammation, further emphasizing its potential as a pharmacological target.

## Discussion

In this study, we show that inhibition of MTH1, using a small molecular compound, decreased the activation of T helper cells in vitro and the allergic inflammatory response in vivo. TH1579 is a drug developed to target nucleotide metabolism by inhibiting the DNA repair enzyme MTH1 (encoded by *Nudt1*). Another study has previously shown that inhibition of a DNA repair enzyme, i.e. 8-oxoguanine DNA glycosylase-1 (OGG1) reduces inflammation and bronchial hyperreactivity in a murine model through interference with the transcriptional activity of NF-kB [17, 18]. However, the anti-inflammatory effect achieved by inhibition of MTH1 is mediated through another mechanism. MTH1 is ubiquitously expressed and has a role to remove ROS-damaged dNTPs from the nucleotide pool, thus preventing the incorporation of oxidized dNTPs into the DNA leading to cellular dysfuntion and malignant transformation. Previously, it has been shown that increased ROS levels, as a consequence of cell proliferation or inflammation, leads to upregulation of MTH1 expression. This has been shown in e.g. tumor cells and activated T cells [9, 19], thus making these MTH1^high^ cells sensitive to pharmacological MTH1 inhibition. In vitro studies using cells from B- and T-cell lymphomas have shown that TH1579 increased the incorporation of oxidized dNTPs into DNA resulting in failure of spindle assembly, prometaphase arrest, and eventually apoptosis [9, 19]. Apoptosis as a consequence of mitotic arrest caused by a defect microtubule-assembly in proliferating cells has been an area of interest in cancer therapy [20]. Similarly, signs of mitotic arrest and increased apoptosis of CD4^+^ T cells was seen in the current study. In a study of psoriasis using human samples and a murine model, Th17-driven inflammation was reduced by TH1827, a close analogue of TH1579. In the current study of OVA-induced allergic inflammation in vivo, the number of all T cell subtypes investigated (CD4^+^, CD8^+^, Th2, and ST2^+^Th2) were reduced following treatment with TH1579. This suggests that inhibition of MTH1 may have a general effect on inflammation driven by T cells, irrespective of the inflammatory profile. However, this warrants further investigation.

In this study, we found that pharmacological inhibition of MTH1 in OVA-induced inflammation dampened several arms of the type 2 immune response. Mice treated with TH1579 displayed markedly reduced total IgE- and OVA-specific- IgE, IgG and IgG1-levels in plasma, responses where Th2 cells have regulating roles [5]. One should bear in mind that, depending on the pathogenesis of allergic disease in mice, the contribution of different antibody subtypes and subclasses to allergic reaction may differ [21]. There are also differences comparing human and murine allergic inflammation. For example, type 2 immune responses IgE, IgG1 (in mice), and IgG4 (in humans) are driven by T cell dependent type 2 immune responses involving IL-4 and IL-13 [22]. A difference was observed in spleen weigth and protein in BAL fluid comparing TH1579 alone with vehicle (data not shown). Since all the groups are sensitized with OVA on two occations it is likely that some systemic inflammation is present. We speculate that TH1579 may reduce this inflammation resulting in the observed differences.

On a cellular level, flow cytometric and histological analysis showed reduced levels of infiltrating immune cells such as T cells and eosinophils, in BAL fluid and lung tissue of mice with OVA-induced inflammation and treatment with TH1579. Even though the numbers of CD4^+^ T cells were reduced in vivo, as well as in vitro, it is unlikely that TH1579 specifically targets only allergen-specific CD4^+^ T cells, but likely MTH1^high^ cells in general. As shown in this study, both the numbers of CD4^+^ and CD8^+^ T cells were reduced in vivo, and the effect of TH1579 on human CD4^+^ and CD8^+^ T cells have previously been investigated in vitro, showing a similar effect [9]. We also investigated the effect of TH1579 treatment in vitro on isolated human CD4^+^ T cells using a different experimental set-up and activation strategy that has been described previously, where CD3^+^ cells were isolated, and pre-activated using a combination of antibodies against CD3 and CD28 conjugated to magnetic beads before treatment [9]. It is likely that these cells are more sensitive to MTH1 inhibition due to the longer activation time, as observed in the increased percentage of apoptotic cells at the same concentration of TH1579. Magnetic beads themselves may also activate cells, which is why we opted for a bead-free CD3/CD28 stimulation. Nevertheless, the result in this study supports the previous in vitro findings where TH1579 promotes apoptosis in activated CD4^+^ T cells through mitotic arrest, while resting T cells are unaffected [9].

Eosinophils are important effector cells in allergic inflammation and possess cytotoxic properties [23]. To investigate possible effect from MTH1-treatment on these cells, human eosinophils were also incubated with TH1579 in the absence and presence of IL-5. IL-5 delays eosinophil apoptosis, and in the absence of IL-5 around 50% of the cells undergo spontaneous apoptosis within two days and within four days most cells are apoptotic which was also confirmed in the current study [24, 25]. Dexamethasone promotes eosinophil apoptosis and was used as a positive control [26, 27]. The addition of TH1579 did not affect the viability of eosinophils in vitro, neither in the absence or presence of IL-5. Eosinophils have the capacity of high ROS production but are terminally differentiated and do not proliferate [23]. This could explain why TH1579 did not affect their viability as opposed to T cells that have both high ROS-production and proliferative capacity. As previously mentioned, the numbers of eosinophils were reduced in TH1579 treated mice. This is likely an indirect effect of MTH1-inhibition, caused by a reduced number of CD4^+^ T cells and consequently, reduced levels of cytokines that promote poesis of eosinophils and their subsequent recruitment to the site of allergic inflammation.

Mast cells are key effector cells in allergic inflammation and cross-linked IgE results in release of a broad set of pro-inflamatory mediators, not least histamine [28]. Interestingly, MTH1-inhibition by TH1579 in mice with OVA-induced inflammation resulted in reduced numbers of mast cells in lung tissue. Recently, a plasticity with regard to mast cell populations have been demonstrated where, in addition to residential yolk-sac derived mast cells, bone-marrow derived mast cells are recruited during inflammation [28, 29]. It is possible that TH1579 affects the proliferation of mast cell progenitors in the bone marrow. However, more likely it is a consequence of the reduced inflammatory response observed in TH1579-treated mice. This warrants further investigation.

Goblet cell hyperplasia and increased mucin production in the airway were observed in mice with OVA-induced inflammation. However, mice treated with TH1579 displayed lower numbers of mucus producing goblet cells in the airways and less MUC5AC production in BAL fluid. *Muc5ac* expression and mucus secretion is enhanced by IL-13 [30, 31]. The reduced levels of IL-13 in BAL fluid, lung tissue homogenate and plasma of TH1579 treated mice, may be one explanation behind the effect on the reduced numbers of goblet cell and mucus secretion.

Currently, TH1579 is used as an oral treatment in phase 1 trials of patients with advanced cancer [10]. In allergic asthma, local administration to the airways, for example nebulized or as a powder, may present advantages, avoiding systemic side effects.

## Conclusions

Taken together, small molecule mediated inhibition of MTH1 using TH1579, reduced allergic airway inflammation in a murine model. TH1579 inhibited T cell proliferation in vitro and reduced the type 2 associated immune response in vivo. These findings suggest that MTH1 could serve as a novel target to treat allergic airway inflammation.

## Electronic supplementary material

Below is the link to the electronic supplementary material.

Supplementary Material 1

Supplementary Material 2

Supplementary Material 3

Supplementary Material 4

Supplementary Material 5

Supplementary Material 6

Supplementary Material 7

Supplementary Material 8

Supplementary Material 9

Supplementary Material 10

Supplementary Material 11

Supplementary Material 12

Supplementary Material 13

Supplementary Material 14

Supplementary Material 15

Supplementary Material 16

Supplementary Material 17

## Data Availability

No datasets were generated or analysed during the current study.
