# Supplementary material for: Pharmacological inhibition of MutT homolog 1 (MTH1) in allergic airway inflammation as a novel treatment strategy
Source: Respir Res. 2025 Mar 14;26:101. doi: 10.1186/s12931-025-03175-z (PMC11909806; doi:10.1186/s12931-025-03175-z)
Supplement: Supplementary file 1 — Supplementary Material 1 [file 12931_2025_3175_MOESM1_ESM.docx]

**Supplementary data**

**Pharmacological inhibition of MutT homolog 1 (MTH1) in allergic airway inflammation as a novel treatment strategy**

Anna Adler^1^, Jesper Bergwik^1^, Médea Padra^1^, Praveen Papareddy^2^, Tobias Schmidt^3,4^, Madelene Dahlgren^5^, Robin Kahn^3,4^, Ulrika Warpman Berglund^6, 7^, Arne Egesten^1^

^1^Division of Respiratory Medicine, Allergology, & Palliative Medicine, Department of Clinical Sciences Lund and Skåne University Hospital, Lund, Sweden, ^2^Department of Laboratory Medicine, Lund University, Lund, Sweden, ^3^Division of Pediatrics Department of Clinical Sciences Lund, Lund University and Skåne University Hospital, Lund, Sweden ^4^Wallenberg Center for Molecular Medicine, Lund, Sweden, ^5^Lung Biology, Department of Experimental Medical Sciences, Lund University, Lund, Sweden ^6^Science for Life Laboratory, Department of Oncology-Pathology, Karolinska Institutet, Solna, Sweden, ^7^Oxcia AB, Stockholm, Sweden.

**Corresponding author**: Dr. Anna Adler; e-mail: Anna.Adler@med.lu.se

1. **MATERIAL AND METHODS**

**1.1 Isolation of human CD4^+^ T cells**

Human whole blood was collected from healthy volunteers in 6 mL Vacutainer-tubes containing sodium heparin (cat#367876; BD, Franklin Lakes, NJ) by venipuncture. Lymphoprep™ (cat#07851; StemCell Technologies, Vancouver, British Columbia) density gradient medium was used according to the manufacturer’s instructions to isolate peripheral blood mononuclear cells (PBMCs). To isolate CD4^+^ T, the EasySep™ Human CD4^+^ T cell isolation kit (cat#17952; StemCell Technologies) together with the EasySep™ magnet (cat#18000; StemCell Technologies) was used as per the manufacturer’s instructions. The cells were counted (XN-350, Sysmex, Kobe, Japan) and purity assessment was performed by flow cytometric analysis. Briefly, 50,000 PBMCs and CD4^+^ T cells were collected from each donor and pelleted by centrifugation at 300 x *g* for 5 min at room temperature (RT). The cell pellets were resuspended in 100 µL of FACS buffer containing 0.5% bovine serum albumin (BSA; Sigma Aldrich, St Louis, MO) in phosphate buffered saline (PBS; Medicago, Uppsala, Sweden) and stained with 1 µL anti-human CD3 antibody-PerCP-Cy5.5 (clone UCHT1; cat# 60011PS.1, StemCell Technologies) and 1 µL anti-human CD4 antibody-APC (clone OKT4; cat#60016AZ.1, StemCell Technologies) for 15 min at RT in the dark. The cells were washed once in FACS buffer and resuspended in a final volume of 200 µL of FACS buffer. The cells were analyzed to check the purity of the isolated cells using the CytoFLEX flow cytometer (Beckman Coulter, Uppsala, Sweden). During the purification process cell numbers were determined using a Sysmex XN-350 (Sysmex Corporation). Whole blood was analyzed using the CBC + DIFF + RET channel profile, and PBMCs and isolated CD4^+^ T cells were counted using only the CBC + DIFF channels.

**1.2 CD4^+^ T cell proliferation**

To follow T cell proliferation 1 x 10^6^ CD4^+^ T cells were stained with 5 µM CellTrace™ Violet (cat#C34557; Invitrogen, Waltham, MA). The cells were resuspended in RPMI-1640 medium supplemented with GlutaΜax-I™ (cat#61870; Gibco/Life Technologies, Carlsbad, CA), 10% fetal calf serum (FBS, cat#10500064, Gibco), 50 U/mL Penicillin Streptomycin (cat#15070-063; Gibco/Life Technologies), and ±25 ng/mL human recombinant IL-2 (CHO-expressed; Cat#78036, StemCell Technologies), and 50,000 cells/well were seeded into a 96-well plate for suspension cultures (cat# 83.3925.500; Sarstedt, Nümbrecht, Germany). The cells were treated with 0.5 µM TH1579 (Oxcia, Stockholm, Sweden) dissolved in DMSO (cat#D2665; Sigma-Aldrich) and activated with 2.5 µL/well ImmunoCult™ Human CD3/CD28 T cell activator (cat#10991; StemCell Technologies). Resting cells and activated cells treated with 0.05% DMSO were used as controls. The cells were incubated for 96 h at 37°C and 5% CO_2_. Resting and activated cells (50,000 cells/sample) from day 0 and day 4 (96 h) from each donor were stained with 1 µL anti-human-CD3-PerCP-Cy5.5 (cat#60011PS.1; StemCell Technologies) and 1 µL anti-human CD25-APC (cat#60158AZ.1; StemCell Technologies) for 15 min at RT protected from light. The cells were pelleted using centrifugation 300 x *g* 5 min at RT and resuspended in FACS buffer. The cells were analyzed for cell proliferation (CellTrace™ Violet) and activation (CD25) using the CytoFLEX flow cytometer (Beckman Coulter).

**1.3 CD4^+^ T cell apoptosis**

Isolated human CD4^+^ T cells were investigated for purity and CD25 expression at 0h and 96h as described above (section 1.2) and seeded in 96-well plates for suspension cultures (cat#83.3925.500; Sarstedt) at two different concentrations, 50,000 and 100,000 cells/well in 100 µL complete cell medium, RPMI-1640 medium supplemented with GlutaΜax-I™ (cat#61870; Gibco/Life Technologies), 10% FBS (cat#10500064; Gibco/Life Technologies) 50 U/mL Penicillin Streptomycin (cat#15070-063; Gibco/Life Technologies), and ±25 ng/mL human recombinant IL-2 (CHO-expressed; Cat#78036; StemCell Technologies), ±2.5 µL/well ImmunoCult™ Human CD3/CD28 T cell activator (cat#10991; StemCell Technologies). The resting and activated cells were then treated with 0.5 or 5 µM TH1579 (Oxcia), dissolved in DMSO (cat#D2650; Sigma-Aldrich) to a final DMSO concentration of 0.05%. Cells treated with 0.05% DMSO was used as a control. The cells were incubated for 96 h at 37°C and 5% CO_2_. The FITC Annexin V Apoptosis Detection Kit I (BD Pharmingen™, cat#556547; BD) was used as per the manufacturer’s instructions. Briefly, 50 µL of sample/well was taken out and washed two times in cold PBS and resuspended in 95 µL 1X Annexin V Binding Buffer and 2.5 µL Annexin V and 2.5 µL propidium iodide (PI). The cells were incubated for 15 min at RT in the dark, after 100 µL 1X Annexin V Binding Buffer was added to each sample, for a final volume of 200 µL/sample. Flow cytometric analysis was then performed to analyze the apoptosis level of the treated T cells in CytoFLEX flow cytometer (Beckman Coulter).

**1.4 Cytospin – human CD4^+^ T cells**

Cytocentrifugation was used to investigate the morphology of the TH1579 treated CD4^+^ T cells. Approximately 25,000 cells/sample (estimated on the seeded concentration) were added to Disposable Epredia™ EZ Single Cytofunnel™ (cat#A78710020; Epredia, Kalamazoo, MI) mounted with a Superfrost Plus Adhesion Microscope slide (cat#J1800AMNZ; Epredia). The final volume was adjusted to 250 µL using PBS. The cytofunnels were placed in a Cytospin 4 (ThermoScientific), and spun for 1000 rpm for 5 min at RT. The microscope slides were left to air dry, and then placed in May Grünwald stain (100 mL May Grünwald stain in 100 mL methanol, ref#01560; HistoLab, Gothenburg, Sweden) diluted in methanol (100 mL) for 10 min at RT. The slides were then rinsed in diH_2_O (3 x 10 dips), and was then added to Giemsa staining solution (20 mL Giemsa solution in 180 mL diH_2_O, cat#01550; HistoLab) for 15 min at RT. The slides were then rinsed in diH_2_O (3 x 10 dips), and left to air dry O/N. The slides were dipped in HistoClear (Cat#14250; HistoLab) and a cover glass was mounted on the slide using a drop of X-tra-Kitt (cat#41-5219-00; Medite, Burgdorf, Germany). The slides were then observed using Nikon Eclipse 80i microscope and images were obtained using the NIS-Elements F3.0 software (Nikon, Tokyo, Japan).

**1.5 Quantification of MTH1 using Jess automated capillary western blot**

Quantitative MTH1 expression in human CD4^+^ T cells (n = 3) was performed in Jess (ProteinSimple, San José, CA), a capillary western blot-based method. Briefly, CD4^+^ T cells were isolated as described in section 1.1, seeded a 12-well plate (Sarstedt) at 0.8 x 10^6^ cells/mL (total volume; 1.25 mL) and cultured with or without CD3/CD28 activation for 96 h. After, 0.5 x 10^6^ cells/sample were collected, washed, and lysed in 300 µL RIPA lysis and extraction buffer (cat#89900; ThermoFisher Scientific) according to the manufacturer’s protocol “lyse suspension-cultured mammalian cells”. Total protein concentration in the samples were determined using Pierce™ BCA Protein Assay Kit (cat#23225; ThermoFisher Scientific) with the microplate procedure according to the manufacturer’s instructions. Jess analysis was performed with the standard settings in the Jess under reducing conditions with chemiluminescent detection according to the manufacturer’s protocol (ProteinSimple). Briefly, cell lysates were diluted with 0.1x sample buffer to a concentration of around 150 µg/mL. After, 4-parts sample was mixed with 1-part 5x fluorescent master mix, containing DTT, and heated for 5 min at 95°C. The prepared samples were loaded to the plate according to the template “immunoassay and total protein” using EZ Standard Pack 1 12-230 kDa (cat#PS-ST01EZ), 12-230 kDa separation capillary cartridges (cat#SM-W004), anti-rabbit detection module (cat#DM-001), total protein detection module (cat#DM-TP01), and RePlex (cat#RP-001) all purchased from ProteinSimple. Rabbit mAb anti-human MTH1 (cat#ab197028, Abcam), diluted 1/100 in antibody diluent 2, was used as the primary detection antibody. Total protein normalization of each sample was performed in the software Compass for Simple Western (version 6.1.0, ProteinSimple). The data is presented as virtual blots with the relative % of total protein (blue dots) in each capillary, and the total peak area of MTH1, detected at 25 kDa ±10%, after total protein normalization ^1^.

**1.6 Isolation of human eosinophils**

Human whole blood was collected from healthy volunteers (n = 3) in 6 mL Vacutainer-tubes containing EDTA (cat#367864; BD) by venipuncture. Eosinophils were isolated using the MACSxpress whole blood eosinophil isolation kit, human (cat#130-104-446; Miltenyi Biotec, Bergisch Gladbach, Germany) as per the manufacturer’s instructions, using 30 mL of human blood/donor as starting material. Sysmex XN-350 was used to track eosinophil purity and cell number during the purification process. The eosinophils were then pelleted and resuspended to a concentration of 375,000 cells/mL in complete cell culture media, RPMI-1640 medium supplemented with GlutaΜax-I™ (cat#61870; Gibco/Life Technologies, Carlsbad, CA), 10% FBS (cat#10500064; Gibco), and 1% Antibiotic–Antimycotic 100X (containing penicillin, streptomycin and Gibco Amphotericin B, cat#12240062, Gibco). Eosinophils, 75,000 cells in 0.2 mL, were seeded into a 96-well plate for suspension cultures (cat#83.3925.500; Sarstedt), ± 10 ng/mL human recombinant IL-5 (cat#200-05; ThermoFisher, Waltham, MA). The cells were treated with 0.5 or 5 µM TH1579 (Oxcia), dissolved in DMSO (cat#D2665, Sigma Aldrich). Untreated eosinophils and eosinophils treated with 0.05% DMSO (cat#D2665; Sigma Aldrich) were used as controls, while 1 µM dexamethasone (cat#D4902; Sigma Aldrich, dissolved in DMSO) treated cells was used as a positive control for apoptosis ^2^. The cells were incubated for 24–96 h at 37°C and 5% CO_2_. At 24, 48, 72 and 96 h approximately 50,000 cells/sample was taken out and the apoptosis levels was analyzed as described above using the FITC Annexin V Apoptosis Detection Kit I (cat#556547; BD Pharmingen™).

**1.7 *In vivo* experiments**

Allergic airway inflammation was induced in BALB/c mice by sensitization with 20 µg of ovalbumin (OVA, cat#vac-pova; EndoFit™, InvivoGen, Toulouse, France) administrated by intraperitoneal injection (i.p.) in 150 µL alum (1:10) on day 0 and 7. On day 14, 16, 18, and 20 the mice were challenged with OVA using intranasal (i.n.) administration of 50 µg OVA (50 µL of 1 mg/mL OVA) dissolved in sterile endotoxin-free water. PBS was used as a negative control. An i.p. injection of either TH1579 (60 mg/kg, Oxcia, Stockholm, Sweden) dissolved in 20% hydroxypropyl-β-cyclodextrins (HPβCD; cat#332607; Sigma-Aldrich, Saint Louis, MI) in acetate buffer (pH 4.5), dexamethasone (2 mg/kg; cat#D4902; Sigma-Aldrich) dissolved in HPβCD (20%), or vehicle (20% of HPβCD) was administered one hour before each OVA challenge. The mice were randomly allocated into five groups, see Supplement Table 1. The mice were sacrificed on day 21 and bronchoalveolar lavage (BAL) fluid, lungs, spleens, and plasma were collected. To collect BAL fluid the trachea of each mouse was cannulated, and lung-lavage was performed three times using a total volume of 1 mL cold PBS. The BAL fluid was kept on ice and then centrifuged (300 x *g*, 5 min at RT), the supernatant was collected for multiplex cytokine analysis (see section 1.13), and stored at –80°C. The pelleted cells were resuspended and analyzed using flow cytometry (see section 1.16).

**Table S1.** Experimental groups of *in vivo* experiments of allergic airway inflammation induced in BALB/c mice. The mice were randomly allocated into five groups: treated with vehicle and OVA challenged (VO, n = 5; initially six mice but one mouse had to be euthanized during the OVA sensitization), treated with TH1579 and OVA challenged (TO, n = 6; n = 5 for BAL fluid samples used in flow cytometric analysis due to technical issues), treated with dexamethasone and OVA challenged (DO, n = 6), treated with TH1579 and PBS challenged (TP, n = 4), and treated with vehicle and PBS challenged (VP, n = 3)

| **Group** | **Number of mice** |
| --- | --- |
| **VO:** Vehicle/OVA | n = 5 |
| **TO:** TH1579/OVA | n = 6 |
| **DO:** Dexamethasone/OVA | n = 6 |
| **TP:** TH1579/PBS | n = 4 |
| **VP:** Vehicle/PBS | n = 3 |

**1.8 Collection of murine lung and spleen tissue**

Lungs and spleens were collected from each mouse and weighed. The left lung from each mouse was placed in 2.5 mL Histofix (cat#01000; Histolab) followed by dehydration in a series of ethanol solutions of increasing concentrations and paraffin embedding. The right lung was split into four pieces. The superior lung lobe was placed in 348 µL T-PER (cat#78510; ThermoFisher Scientific) containing 2 µL of a protease and phosphatase inhibitor cocktail (Halt™, cat#78440; ThermoFisher Scientific). The samples were stored at –80°C until used for multiplex cytokine immunoassay and protein analysis (see section 1.12). The middle lobe and inferior lobe were placed in PBS for immediate homogenization and flow cytometry analysis (see sections 1.11 and 1.15). The post-caval lobe was submerged in 350 µL of RNAlater (cat#AM7021; ThermoFisher Scientific) and stored at –80°C until used for qPCR (see sections 1.17, 1.18, 1.22).

**1.9 Histology**

From each lung tissue sample embedded in paraffin blocks several cm^2^-size, 4 µm thick, paraffin sections were cut with a microtome (RM2255; Leica, Wetzlar, Germany) and the sections were placed on Superfrost Plus Adhesion microscope slides (cat. #J1800AMNZ; Epredia) for staining.

**1.10** **H&E staining**

Pathological changes in lung tissues were assessed with hematoxylin and eosin (H&E) staining. Briefly, paraffin embedded lung tissue sections (4 µm) mounted on microscopic glass slides were baked at 65°C for 30 min to remove excess paraffin. Thereafter, the slides were further deparaffinized by treating the slides in two successive Histolab-Clear (ref#14250; HistoLab) baths, 4 min each. The lung sections were then hydrated by passing the slides through alcohol baths with decreasing alcohol concentrations; absolute alcohol for 4 min, absolute alcohol for 4 min, 95% alcohol for 4 min and finally 70% alcohol for 4 min. The slides were then washed in tap water for 1 min followed by staining with Mayer’s hematoxylin stain (cat#01820; HistoLab) for 10 min followed by washing in running tap water for 10 min. The slides were then rinsed in distilled water and counterstained with 0.2% eosin (cat#10650; HistoLab) for 5 min, followed by a quick rinse in distilled water. The slides were then dehydrated by passing the slides through alcohol baths with increasing alcohol concentrations; 70% alcohol for 30 seconds, 95% alcohol for 30 seconds, absolute alcohol for 30 seconds, absolute alcohol for 5 min followed by two successive Histolab-Clear (Cat#14250; HistoLab) baths for 3 min each. Cover slips were then mounted on top of the H&E-stained lung tissue sections by applying a drop of X-Tra-Kitt (cat#415219-00; Medite) mounting medium and carefully placing a cover slip glass on top. All the staining and washing steps were performed at RT. The slides were left to dry O/N. After, the slides were scanned with Aperio ScanScope CS scanner (Leica Biosystems) at 20x magnification. Areas of inflammatory cell infiltrates in H&E stained lung sections were quantified using computer-assisted morphometrical analysis (QuPath). The measured inflammatory area values were normalized to the whole area of each tissue section.

**1.11 PAS staining**

Airway mucus secretion and goblet cells in lung tissue were assessed with PAS staining. Paraffin embedded lung tissue sections (4 µm) mounted on microscopic glass slides were baked, deparaffinized and hydrated as described above. After, the slides were immersed in periodic acid solution (PAS Stain kit, cat#62328; Abcam, Cambridge, UK) for 5 min. The slides were rinsed in four changes of diH_2_O. Then, the slides were immersed in 25% Schiff’s solution (Schiff’s reagent Cat#3952016, Sigma-Aldrich), 50 mL diluted in 150 mL MQ-H_2_O for 15 min. The slides were then washed in running hot water followed by rinsing in diH_2_O. This was followed by staining with Mayer’s hematoxylin stain (cat#01820; HistoLab) for 3 min. The slides were then rinsed in running water, followed by a quick rinse in diH_2_O. The slides were then dehydrated, and cover slips were mounted as described above. The slides were left to dry O/N. All the staining and washing steps were performed at RT. Mucus production was analyzed by calculating PAS positive cells (%) in the airways using computer-assisted morphometrical analysis (QuPath). The average percentage of PAS positive cells in four airways per tissue section was measured.

**1.12 Lung tissue homogenization for cytokine and protein analysis**

The superior lung lobe collected for cytokine and protein analysis was thawed and homogenized with 1.4 mm ceramic beads (cat#13113-325; Qiagen, Hilden, Germany) in the MagNA Lyser Instrument (Roche, Mannheim, Germany) at 6,000 rpm for 30 seconds. The lung homogenates were centrifuged at 15,000 x *g* for 10 min at 4°C and the supernatants were collected and stored at –80°C until cytokine– and protein analysis.

**1.13 BioPlex cytokine analysis**

A Bio-Plex Pro mouse cytokine assay (cat#M60009RDPD; 23-Plex Group I; Bio-Rad, Hercules, CA) using a Luminex-xMAP/Bio-Plex 200 System was used to quantify multiple cytokines in plasma, BALF and lung homogenate. Analysis was performed using Bio-Plex Manager 6.2 software (Bio-Rad). The detection limits/the standard curves were as follows: IL-1α (9338.00-0.57 pg/mL), IL-1β (23194.00-1.42 pg/mL), IL-2 (21349.00-1.30 pg/mL), IL-3 (10754.00-0.66 pg/mL), IL-4 (8854.00-0.54 pg/mL), IL-5 (15187.00-0.93 pg/mL), IL-6 (11020.00-0.67 pg/mL), IL-9 (44099.00-2.69 pg/mL), IL-10 (61796.00-3.77 pg/mL), IL-12p40 (87778.00-5.36 pg/mL), IL-12p70 (32223.00-1.97 pg/mL), IL-13 (214865.00-13.11 pg/mL), IL-17 (8686.00-0.53 pg/mL), CCL11 (Eotaxin) (17740.00-1.08 pg/mL), GCSF (98665.00-6.02 pg/mL), GMCSF (76779.00-4.69 pg/mL), IFN-γ (15753.00-0.96 pg/mL), KC (24609.00-1.50 pg/mL), MCP-1 (260889.00-15.92 pg/mL), MIP-1α (4289.00-0.26 pg/mL), MIP-1β (36551.00 pg/mL), RANTES (62700.00-3.83 pg/mL), and TNF-α (59236.00-3.62 pg/mL). Correction for protein concentrations in the lung homogenate was done using a Pierce™ BCA Protein Assay Kit (Cat#23225; ThermoFisher Scientific), see section 1.13. The BioPlex assays were performed according to the manufacturers protocol with the exception that a final volume for the analysis was 25 µL. The BAL fluid was analyzed undiluted, whereas the plasma and lung homogenate were diluted 1:2 in sample diluent.

**1.14 Total protein concentration**

The total protein concentration in BAL fluid was determined using Pierce™ BCA Protein Assay Kit (cat#23225; ThermoFisher Scientific,) using the microplate procedure according to the manufacturer’s instructions. Briefly, 25 µL BAL fluid (undiluted) was added in duplicates to a 96-well microtiter plate, together with the diluted standards. The working reagent (WR) was prepared, and 200 µL WR was added to each well. The plate was incubated at 37°C for 30 min, and the absorbance was measured at 550 nm using an iMark Microplate reader (Bio-Rad). A standard curve was generated (Sigmoidal, 4 PL) and used to calculate the total protein levels in the samples. The protein concentration in the homogenized lungs were determined using the same method and protocol, with the exception that the samples were diluted 1:10 in PBS.

**1.15 Lung tissue homogenization for flow cytometry**

The lung pieces collected for flow cytometry were dissociated using the Mouse Lung Dissociation kit (cat#130-095-927; Miltenyi Biotec) per manufacturers’ instructions. The lung pieces were placed into gentleMACS™ C tubes (one tube/lung piece) (cat#130-093-237; Miltenyi Biotec) containing the enzyme mix described in the protocol of the Mouse Lung Dissociation kit. The gentleMACS™ C tubes were placed onto the gentleMACS octo dissociator (Miltenyi Biotec), using the 37C_m_LDK_1 program after attaching the heating elements. Following completion of the program, the m_lung_02 program was run. After, the lung homogenates were filtered through a 70 µm cell strainer (cat#431751; Corning, USA) into a 50 mL Falcon tube. The cell suspensions were centrifuged at 300 x *g* for 10 minutes, and the supernatant was removed. The remaining erythrocytes were lysed by hypotonic lysis in 2.5 mL sterile MQ-H_2_O for 20 seconds followed by the addition of 2.5 mL 1.8% NaCl to restore the solution to an isotonic 0.9% NaCl solution. The cell suspensions were then centrifuged at 300 x *g* for 5 minutes and thereafter resuspended in 100 µL FWB buffer (PBS without Mg^2+^ and Ca^2+^) (cat#14190-144; Gibco), 2% FBS (cat#10500064; Gibco) and 1 mM EDTA (cat#1861274, ThermoFisher Scientific), and 40 µL were taken out for flow cytometric analysis (see section 1.16).

**1.16 Flow cytometry**

Cell suspensions (from BALF and lung tissue homogenate) were resuspended in 50 µL 5 µg/mL mouse BD Fc-block™ (Rat anti-mouse CD16/CD32, 2.4G2, cat#553142; BD Pharmingen) in FWB and incubated for 30 min. After, 100 µL PBS was added to each sample and the cells were washed once at 300 x *g* for 3 min. The supernatant was discarded. After, 50 µL of Live/Dead™ Aqua stain (cat#L34965; Invitrogen, Waltham, MA) diluted 1/1000 in PBS, was added to each sample and incubated for 20 min at RT protected from light. The cells were pelleted (300 x *g* for 3 min) and the supernatant was discarded. Next, of the following antibodies were used for staining at a concentration of 1 µg/mL diluted in FWB for 45 min protected from light: FITC-CD45 (cat#553080; BD), PE-CD3 (cat#555275; BD), PEDazzle594-CD19 (cat#11554; BioLegend), PEDazzle594-CD11b (cat#101256; BioLegend), BV421-ST2 (cat#145309; BioLegend), BV605-TCRγδ (cat#118129; BioLegend), BV50-CD8a (cat#100742; BioLegend), BV785-CD4 (cat#100552; BioLegend), APC-Cy7-CD49b (cat#108920; BioLegend), AF700-Thy1.1 (cat#202528; BioLegend), AND BV650-Siglec-F (cat#740557; BD). Fοr more information about the antibodies, see Supplement Table 2. After, 100 µL FWD was added to each sample, and the cells were pelleted at 300 x *g* for 3 min. The supernatant was discarded, and the cells were fixed and permeabilized in 200 µL FoxP3 Fixation/Permeabilization working solution from the eBioscience™ Foxp3/Transcription factor staining buffer set (cat#00-5523; Invitrogen) O/N at 4°C. After, the cells were pelleted by centrifugation at 400 x *g* for 3 min. The supernatant was discarded, and the cell pellet was resuspended in 200 µL 1X Permeabilization buffer from the Foxp3 staining buffer set. The cells were washed in total two times in 200 µL 1X Permeabilization buffer. The following antibodies were used for the intracellular staining diluted in 1X Permeabilization buffer at a concentration of 3 µg/mL: PerCP-Cy5.5-FoxP3 (cat#46-5773-82; Invitrogen), PE-Cy7-Rorγt (cat#25-6981-82; Invitrogen) and APC-Gata3 (cat#50-9966-42; Invitrogen). The samples were incubated for 45 min protected from light. After, 100 µL 1X Permeabilization buffer was added, and the cells were centrifuged at 400 x *g* for 3 min. One additional washing step was performed with 200 µL 1X Permeabilization buffer. The cells were pelleted at 400 x *g* for 3 min, and the BAL fluid samples were resuspended in 150 µL FWB, whereas the lung tissue samples were resuspended in 300 µL FWB. The samples were stored at 4°C protected from light until flow cytometric analysis using the Fortessa/X20 (BD). Right before analysis 5 µL of CountBright™ Absolute counting beads (5,200 beads/sample, cat#C36950; Invitrogen) was added to each sample and used to estimate the number of cells in the samples. Additionally, before analysis of lung tissue samples, they were strained through 5 mL Falcon™ Round-bottom Polystyrene Test tubes with cell strainer snap cap with 35 µm mech (cat#352235; Falcon™). FMO-1 controls were prepared using pooled cells from the single cell suspensions obtained from the homogenized lung tissue samples. Single stain control for the Live/Dead Aqua stain was prepared using ArC™ amine reactive compensation bead kit (cat#A10628; Invitrogen) according to the manufacturer’s instructions. Whereas single stain controls for the antibodies were prepared using Ultracomp eBeads (cat#01-2222-41; Invitrogen) according to the manufacturer’s protocol.

**Table S2.** Antibody panel used for flow cytometric analysis of bronchoalveolar lavage (BAL) fluid collected from the OVA-sensitized and challenged mice.

|  | **Color** | **Target (mouse)** | **Concentration** | **Per 1 mL** | **Host species** | **Specifications** |
| --- | --- | --- | --- | --- | --- | --- |
| **1** | BV510 | Live/Dead Aqua |  | 1 µL/mL in PBS |  | Invitrogen; Cat#L34965 |
| **2** | FITC | CD45 | 0.5 mg/mL | 2 µL | Rat | BD; Cat#553080 |
| **3** | PE | CD3 | 0.2 mg/mL | 5 µL | Rat | BD; Cat#555275 |
| **4** | PEDazzle594 | CD19 | 0.2 mg/mL | 5 µL | Rat | BioLegend; Cat#11554 |
| **5** | PEDazzle594 | CD11b | 0.2 mg/mL | 5 µL | Rat | BioLegend; Cat#101256 |
| **6** | BV421 | ST2 (IL-33Ra) | 0.2 mg/mL | 5 µL | Rat | BioLegend; Cat#145309 |
| **7** | BV605 | TCRgd | 0.2 mg/mL | 5 µL | Hamster | BioLegend; Cat#118129 |
| **8** | BV650 | CD8a | 0.2 mg/mL | 5 µL | Rat | BioLegend; Cat#100742 |
| **9** | BV785 | CD4 | 0.2 mg/mL | 5 µL | Rat | BioLegend; Cat#100552 |
| **10** | APC-Cy7 | CD49b | 0.2 mg/mL | 5 µL | Rat | BioLegend; Cat#108920 |
| **11** | AF700 | Thy1.1 (CD90.1) | 0.5 mg/mL | 2 µL | Mouse | BioLegend; Cat#202528 |
| **12** | BV650 | Siglec-F | 0.2 mg/mL | 5 µL | Mouse | BD; Cat#740557 |
| **13** | PerCP-Cy5.5 | FoxP3 | 0.2 mg/mL | 15 µL | Rat | Invitrogen; cat#46-5773-82 |
| **14** | PE-Cy7 | Rorγτ | 0.2 mg/mL | 15 µL | Rat | Invitrogen; Cat#25-6981-82 |
| **15** | APC | Gata3 | 0.025 mg/mL | 15 µL | Rat | Invitrogen; Cat#50-9966-42 |

**1.17 Lung tissue homogenization for RT-PCR**

The post-caval lobe collected for mRNA extraction was homogenized in 600 µL Buffer RLT (from the RNeasy Mini Kit, Qiagen, Valencia, CA) with β-mercaptoethanol (cat#436022A; VWR, Radnor, PA) using 1.4 mm ceramic beads (cat#13113-325; Qiagen). The tubes were then placed in a MagNA Lyser Instrument (Roche, Basel, Switzerland) at speed of 6,000 rpm for 30 seconds to homogenize the lung tissue. The lung homogenates were centrifuged at 15,000 x *g* for 10 min at 4°C. The supernatants were collected and stored at –80°C until mRNA extraction.

**1.18 Real-time PCR Array**

Total mRNA was extracted from homogenized lung tissue (described above) using the RNeasy® Mini Kit (cat#74106; Qiagen) according to the manufacturer’s protocol. The RNA concentrations were determined with a NanoDrop ND1000 (Saveen Werner, Malmö, Sweden). Equal amounts of RNA were pooled from several animals in each group (OVA/vehicle n = 5, OVA/TH1579 n = 6, OVA/dexamethasone n = 6, PBS/TH1579 n = 4, and PBS/vehicle n = 3). RNA was converted into cDNA (500 ng) using an iScript™ Advanced cDNA Synthesis Kit (cat#1708890; Bio-Rad) according to the manufacturer’s protocol. The cDNA reaction was performed using the C1000 Touch Thermal Cycler (Bio-Rad) using the following settings: priming (5 min at 25°C), reverse transcription (20 min at 46°C), RT reaction (1 min at 95°C). Thereafter, the resulting cDNA was mixed with RT^2^ Syber® Green ROX™ qPCR Mastemix. A volume of 25 µL of the reaction mix was added to each well of a RT^2^ Profiler™ PCR Array Mouse Allergy & Asthma PAMM-067ZA plate (Qiagen). The RT-PCR reaction was performed using a Quantstudio™ 7 Flex system (ThermoFisher Scientific) and data analysis were performed using the manufacturer’s web-based software (<https://geneglobe.qiagen.com/analyze>). Normalization of gene expression was performed using the following house-keeping genes: *B2m*, *Actb*, *Gusb*, *Gapdh* and *Hsp90ab1*.

**1.19 Total IgE and OVA-specific IgE ELISA**

Total IgE levels in plasma were determined using a IgE mouse ELISA kit (cat#EMIGHE; Invitrogen). The ELISA was performed according to the manufacturer’s instructions. The plasma from VO (vehicle-treatment/OVA-induced inflammation), TO (TH1579-treatment/OVA-induced inflammation) and DO (dexamethasone-treatment/OVA-induced inflammation) groups were diluted 1:2,000 and the VP and TP groups were diluted 1:10 to fit within the range of the standard curve. The standard curve ranged from 100.0-0.14 ng/mL of IgE. The absorbance was measured at 450 nm using a VICTOR 1420 Multilabel plate reader (PerkinElmer, Waltham, MA). A standard curve was generated (Sigmoidal, 4 PL, GraphPad) and used to calculate the IgE levels in the samples. The mouse anti-OVA serum IgE antibody detection ELISA kit (cat#3010; Chondrex, Woodinville, WA) for serum and plasma samples was used to measure the OVA-specific IgE levels in plasma. The ELISA was performed as per the manufacturer’s instructions. The standard curve ranged from 25.0-0.4 ng/mL OVA-specific IgE, and the plasma samples were diluted 1:10. The absorbance was measured at 450 nm using an iMark Microplate reader (Bio-Rad). A standard curve was generated (four-parameter logistic curve) and used to calculate the OVA specific IgE-levels in the plasma samples.

**1.20 Total IgG, OVA-specific IgG and IgG1 ELISA**

The total IgG-levels in plasma were measured using the IgG (total) mouse uncoated ELISA kit with plates from Invitrogen (cat#88-50400-22). Samples were diluted 1:1,000 and the assay was performed as per the manufacturer’s instructions. The standard curve ranged from 100.0-1.6 ng/mL IgG. The mouse anti-OVA IgG antibody subtype/subclass ELISA (cat#3011; Chondrex) and the mouse anti-OVA IgG1 antibody subtype/subclass ELISA (cat#3013, Chondrex) for serum and plasma samples was used to measure the OVA-specific IgG and IgG1 levels in plasma. The ELISAs were performed according to the manufacturer’s instructions and the samples were diluted 1:100,000 for both ELISAs. The standard curves for OVA-specific IgG and IgG1both ranged from 12.5-0.2 ng/mL. The absorbance was measured at 450 nm using an iMark Microplate reader (Bio-Rad). A standard curve was generated (four-parameter logistic curve) and used to calculate the total IgG-, OVA-specific IgG and OVA-specific IgG1 in the plasma samples.

**1.21 MUC5AC ELISA**

To analyze the amount of mucin-5 subtype AC (MUC5AC) in collected BAL fluid samples, a MUC5AC ELISA kit (cat#MBS2507150; MyBioSource, San Diego, CA) was used as per the manufacturer’s instructions. The standard curve ranged from 10.0-0.16 ng/mL MUC5AC, and to fit within the standard curve the VO, TO and DO samples were diluted 1:30, while TP and VP were diluted 1:5. The absorbance was measured at 450 nm using an iMark Microplate reader (Bio-Rad). A standard curve was generated (four-parameter logistic curve) and used to calculate the MUC5AC-levels in the samples.

**1.22 Analysis of mRNA expression levels of *Nudt1* using RT-qPCR**

A two-step quantitative reverse transcriptase-polymerase chain reaction (RT-qPCR) was used to determine the mRNA expression of *Nudt1* and the housekeeping gene *Gapdh*. Total RNA was purified from murine lung tissue as described in sections 2.16 and 2.17. The High Capacity cDNA Reverse Transcription kit (cat#4368814; Applied Biosystems, ThermoFisher Scientific, Vilnius, Lithuania) was used to obtain cDNA by reverse transcription of the isolated RNA from the Vehicle/OVA and PBS/OVA groups. cDNA synthesis less than 2 µg of total RNA was used as recommended by Applied Biosystems. TaqMan™ Fast Advance Master Mix (cat#4444557; Applied Biosystems) was used for multiplex quantification the mRNA levels of *Nudt1* and *Gapdh* using the following commercially synthesized probes from Applied Biosystems:

*Nudt1*: VIC-MGB 360 rxns, Assay ID Mm00599710_m1;

*Gapdh*: 6FAM™-MGB, 2500 rxns, Assay ID Mm99999915_g1.

The qPCRs were performed in 96-well standard (0.2 mL) plates, in duplicates on the QuantStudio™ 7 Flex Real-Time PCR System (Applied Biosystems). The volumes of reagents per reaction were as follows; 10.0 µL TaqMan™ Fast Advance Master Mix (2X), 1 µL of each of the probes, 7.0 µL nuclease-free water and 2 µL of the cDNA preparation. Quantification of *Nudt1* was done relative to *Gapdh* reference RNA using the relative 2^-ΔCt^ method.

**1.23 Toluidine Blue staining**

The number of mast cells in lung tissue was assessed using toluidine blue staining. Paraffin embedded lung tissue sections (4 µm) mounted on microscopic glass slides were baked, deparaffinized and hydrated as described above. The slides were then stained with a mixture of 20 mL toluidine blue (cat#89640; Sigma-Aldrich) dissolved in 70% ethanol which was added 180 mL 1% sodium chloride (pH 2.2) for 3 min at RT. The slides were then rinsed in running water, followed by a quick rinse in diH_2_O. The slides were then dehydrated and cover slips were mounted as described above (section 1.9). The slides were left to dry O/N. All the staining and washing steps were performed at RT. All mast cells in each section were then counted manually using Nikon Eclipse 80i microscope (Nikon). The number of mast cells per tissue was normalized to tissue size which was measured in QuPath. Images were obtained using the NIS-Elements F3.0 software (Nikon).

**1.24 *In vivo* pilot study – lower dose TH1579**

Allergic airway inflammation was induced in BALB/c mice by sensitization with 20 µg of ovalbumin (OVA, cat#vac-pova; EndoFit™, InvivoGen, Toulouse, France) administrated by intraperitoneal injection (i.p.) in 150 µL alum (1:10) on day 0 and 7. On day 14, 15 and 16 the mice were challenged with OVA using intranasal (i.n.) administration of 50 µg OVA (50 µL of 1 mg/mL OVA) dissolved in sterile endotoxin-free water. PBS was used as a negative control. An i.p. injection of either TH1579 (45 mg/kg) dissolved in 20% hydroxypropyl-β-cyclodextrins (HPβCD; cat#332607; Sigma-Aldrich) in acetate buffer (pH 4.5), dexamethasone (3 mg/kg; cat#D4902; Sigma-Aldrich) dissolved in HPβCD (20%), or vehicle (20% of HPβCD) was administered one hour before each OVA challenge. The mice were randomly allocated into five groups: treated with vehicle and OVA challenged (VO, n = 8), treated with TH1579 and OVA challenged (TO, n = 8), treated with dexamethasone and OVA challenged (DO, n = 8), treated with TH1579 and PBS challenged (TP, n = 8), and treated with vehicle and PBS challenged (VP, n = 8). The mice were sacrificed on day 17 and lungs, spleens, plasma and bronchoalveolar lavage (BAL) fluid were collected, and analyzed for plasma antibody levels, total protein levels in BAL fluid, inflammatory cell infiltration using flow cytometry and lung histology using H&E staining. The experiments were performed as described in sections 7**-**10 and 1.19, with the exceptions that CytoFLEX (Beckman Coulter) was used for the flow cytometric analysis, and a four-point manual scoring scale (0-3; 0 = normal; 1 = mild inflammation involving the peribronchial or perivascular area; 2 = intermediate inflammation involving less than 50% parenchyma; 3 = severe inflammation involving more than 50% parenchyma), was used to determine the grade of lung inflammation, by an independent blinded researcher, in H&E-stained lung sections ^3,4^.

**1.25 Software**

Graphical illustrations of experimental procedures were created using BioRender (BioRender.com). QuPath software (version v0.4.3) was used for the histological analyses, FlowJo (version 10.9.0, Becton Dickinson & Company) was used for flow cytometric analyses, and Compass for Simple Western (version 6.1.0, ProteinSimple) was used for Jess analyses.

1. **SUPPLEMENTARY FIGURES**

**Figure S1.** Representative histograms showing the expression of the surface marker CD25 on isolated CD4^+^ T cells seeded at 100,000 cells/well in 100 µL complete cell culture medium, after 96 h ±CD3/CD28 stimulation and ±TH1579 treatment or 0.05% DMSO (n = 6). CD25 is upregulated upon CD3/CD28 stimulation, however it is less expressed on TH1579 treated cells.

**Figure S2.** Representative experiment showing the gating strategy of resting and CD3/CD28 stimulated T cells to determine apoptosis levels in CD4^+^ T cells using Annexin V-FITC and propidium iodine (PI) staining.

**Table S3.** Summary of CD4^+^ T cell apoptosis data. TH1579 induce apoptosis in CD3/CD28 stimulated human CD4^+^ T cells. Statistical comparisons were performed using one-way ANOVA with Dunnett’s post hoc test comparing the mean of each group to the group treated with 0.05% DMSO (*****P*<0.0001, ****P*<0.001, ***P*<0.01, **P*<0.05).

**Figure S3A–G.** Summary of apoptosis data in % of isolated human eosinophils cultured at 37°C and 5% CO_2_ following treatment with 0.05% DMSO (negative control), 0.5 µM TH1579, 5 µM TH1579 or 1 µM dexa/dexamethasone (positive control). **(A)** 24 h treatment, **(B)** 24 h treatment + IL-5, **(C)** 96 h treatment and **(D)** 96 h treatment + IL-5. **(E)** Representative flow cytometry dot plots of double stained Annexin V-FITC/Propidium iodine (PI) eosinophils treated with 1 µM dexamethasone (positive control) ±IL-5 (n = 3). **(F-I)** Viability of eosinophils in % post treatment for 24-96 h, as determined with Annexin V-FITC and PI apoptosis kit. Statistical analysis was preformed using a paired t-test between 1 µM dexamethasone and 5 µM TH1579. The results are displayed as mean ± SD (*****P*<0.0001, ****P*<0.001, ***P*<0.01, **P*<0.05). **(J)** Representative images of May Grünwald-Giemsa stained cytospins (20x, scale bar = 5 µm) of human eosinophils 48 h after exposure to 1 µM dexamethasone or 5 µM TH1579 ±IL-5. Apoptotic eosinophils display morphological changes of shrinkage, membrane blebbing, nuclear condensation, darkening of cytoplasmic staining ^5^. Annexin V-FITC and PI apoptosis analysis and morphological examination of dexamethasone treated eosinophils clearly displays disrupted/apoptotic cells, while IL-5 treatment effectively inhibits the eosinophils from entering an apoptotic state. While no difference was observed between 0.05% DMSO or 0.5/5 µM TH1579 treated eosinophils, thus TH1579 did not induce apoptosis in human eosinophils *in vitro*.

**Figure S4A-E. (A)** Overview of the experimental design of the female BALB/c mice model of ovalbumin (OVA)-induced allergic airway inflammation. **(B)** Lung weights, vehicle/OVA (VO, n = 8), TH1579/OVA (TO, n = 8), dexamethasone/OVA (DO, n = 8), TH1579/PBS (TP, n = 8), and vehicle/PBS (VP, n = 8). **(C)** Total protein concentration in the bronchoalveolar lavage (BAL) fluid. **(D)** Quantification of OVA-specific IgE in plasma (VO; n = 7, TO; n = 8, DO; n =3, TP; n = 3, VP; n = 3). **(E)** Haemotoxylin and eosin (H&E)- staining of murine lung tissue sections from OVA-challenged mice with and without 45 mg/kg TH1579 treatment. **(F)** Representative images of H&E-stained lungs. Scale bar: 10 µm, 10x magnification. Results are displayed as mean ± SD, statistical comparisons were performed using one-way ANOVA with Dunnett’s post hoc test (*****P*<0.0001, ****P*<0.001, ***P*<0.01, **P*<0.05).

**Figure S5.** Quantification of inflammatory cells in bronchoalveolar lavage (BAL) fluid and homogenized lung tissue using flow cytometric analysis. Vehicle/OVA (VO, n = 8), TH1579/OVA (TO, n = 8), dexamethasone/OVA (DO, n = 8), TH1579/PBS (TP, n = 8), and vehicle/PBS (VP, n = 8). The results are displayed as mean ± SD, statistical comparisons were performed using one-way ANOVA with Dunnett’s post hoc test (*****P*<0.0001, ****P*<0.001, ***P*<0.01, **P*<0.05).

**Figure S6A and B.** A representative experiment showing the gating strategy of viable leukocytes (Live/Dead Aqua^–^CD45^+^), T cells (Live/Dead Aqua^–^CD45^+^CD3^+^), T helper cells (Live/Dead Aqua^–^CD45^+^CD3^+^CD4^+^), cytotoxic T cells (Live/Dead Aqua^–^CD45^+^CD3^+^CD8^+^), Th2 cells (Live/Dead Aqua^–^CD45^+^ CD3^+^CD4^+^GATA3^+^), ST2+ Th2 cells (Live/Dead Aqua^–^CD45^+^CD3^+^CD4^+^GATA3^+^ST2^+^), Tregs (Live/Dead Aqua^–^CD45^+^CD3^+^CD4^+^FoxP3^+^) and eosinophils (Live/Dead Aqua^–^CD45^+^CD11b^+^Siglec-F^+^) in **(A)** bronchoalveolar lavage (BAL) fluid and **(B)** homogenized lung tissue collected from OVA-challenged mice with and without 45 mg/kg TH1579 treatment.

**Figure S7A–F.** Scanned sections hematoxylin and eosin (H&E) stained of lungs collected from the OVA-sensitized and challenged mice. **(A)** Vehicle/OVA, **(B)** TH1579/OVA, **(C)** Dexamethasone/OVA, **(D)** TH1579/PBS and **(E)** Vehicle/PBS. **(F)** Example how H&E-stained sections were evaluated in QuPath. The brush tool in QuPath were used to manually mark the area of inflammatory lesions (red). The software annotation calculated the sum of the total area (µm^2^) marked as inflammatory lesions in each lung section.

**Figure S8A–F.** Scanned sections of periodic acid-Schiff (PAS)-stained lungs collected from the OVA-sensitized and challenged mice. **(A)** Vehicle/OVA, **(B)** TH1579/OVA, **(C)** Dexamethasone/OVA, **(D)** TH1579/PBS and **(E)** Vehicle/PBS. **(F)** Example of how the PAS-stained tissue sections were evaluated in QuPath. The area of the epithelial cell layer was marked with the brush tool (yellow). The PAS positive (red) and negative cells (blue) were classified, and the PAS positive cells within the marked (yellow) area were counted by the positive cell count annotation of the software. The % of PAS positive cells per airway was calculated by the software.

**Figure S9A–D. (A)** Quantification of inflammatory cells in homogenized lung tissue using flow cytometric analysis. Vehicle/OVA (VO, n = 5), TH1579/OVA (TO, n = 6), dexamethasone/OVA (DO, n = 6), TH1579/PBS (TP, n = 4), and vehicle/PBS (VP, n = 3). Representative dot plots of; **(B)** type 2 helper (Th2) cells (Live/Dead Aqua^–^CD45^+^ CD3^+^CD4^+^GATA3^+^) and ST2^+^Th2 cells (Live/Dead Aqua^–^CD45^+^CD3^+^CD4^+^GATA3^+^ST2^+^), **(C)** type 2 innate lymphoid cells (Live/Dead Aqua^–^CD45^+^CD3^–^GATA3^+^ST2^+^), and **(D)** eosinophils (Live/Dead Aqua^–^CD45^+^CD11b^+^Siglec-F^+^). The results are displayed as mean ± SD, statistical comparisons were performed using one-way ANOVA with Dunnett’s post hoc test (*****P*<0.0001, ****P*<0.001, ***P*<0.01, **P*<0.05).

**Figure S10A and B.** A representative experiment showing the gating strategy of viable leukocytes (Live/Dead Aqua^–^CD45^+^), T cells (Live/Dead Aqua^–^CD45^+^CD3^+^), T helper cells (Live/Dead Aqua^–^CD45^+^CD3^+^CD4^+^), cytotoxic T cells (Live/Dead Aqua^–^CD45^+^CD3^+^CD8^+^), ILC2s (Live/Dead Aqua^–^CD45^+^CD3^–^GATA3^+^ST2^+^), Th2 cells (Live/Dead Aqua^–^CD45^+^CD3^+^CD4^+^GATA3^+^), ST2^+^ Th2 cells (Live/Dead Aqua^–^CD45^+^CD3^+^CD4^+^GATA3^+^ST2^+^), and eosinophils (Live/Dead Aqua^–^CD45^+^CD11b^+^Siglec-F^+^) in **(A)** bronchoalveolar lavage (BAL) fluid and **(B)** homogenized lung tissue collected from OVA-challenged mice with and without TH1579 treatment.

**Figure S11.** Multiplex immunoassay of bronchoalveolar lavage (BAL) fluid. Individual graphs of all 23 cytokines measured in BAL fluid from mice after OVA-induced airway inflammation. Vehicle/OVA (VO, n = 5), TH1579/OVA (TO, n = 6), dexamethasone/OVA (DO, n = 6), TH1579/PBS (TP, n = 4), and vehicle/PBS (VP, n = 3). The results are displayed as mean ± SD, statistical comparisons were performed using one-way ANOVA with Dunnett’s post hoc test (*****P*<0.0001, ****P*<0.001, ***P*<0.01, **P*<0.05).

**Figure S12.** Individual graphs of the 23 cytokines measured in lung tissue homogenate from mice after OVA-induced airway inflammation using a multiplex immunoassay. Vehicle/OVA (VO, n = 5), TH1579/OVA (TO, n = 6), dexamethasone/OVA (DO, n = 6), TH1579/PBS (TP, n = 4), and vehicle/PBS (VP, n = 3). The results are displayed as mean ± SD, statistical comparisons were performed using one-way ANOVA with Dunnett’s post hoc test (*****P*<0.0001, ****P*<0.001, ***P*<0.01, **P*<0.05).

**Figure S13.** Individual graphs of all 15 out of 23 measurable cytokines in plasma from mice after OVA-induced airway inflammation using a multiplex immunoassay. Vehicle/OVA (VO, n = 5), TH1579/OVA (TO, n = 6), dexamethasone/OVA (DO, n = 6), TH1579/PBS (TP, n = 4), and vehicle/PBS (VP, n = 3). The results are displayed as mean ± SD, statistical comparisons were performed using one-way ANOVA with Dunnett’s post hoc test (*****P*<0.0001, ****P*<0.001, ***P*<0.01, **P*<0.05).

1. **REFERENCES**

1 Collins, J. M. & Wang, D. Cytochrome P450 3A4 (CYP3A4) protein quantification using capillary western blot technology and total protein normalization. *J Pharmacol Toxicol Methods* **112**, 107117 (2021). <https://doi.org:10.1016/j.vascn.2021.107117>

2 Debierre-Grockiego, F., Leduc, I., Prin, L. & Gouilleux-Gruart, V. Dexamethasone inhibits apoptosis of eosinophils isolated from hypereosinophilic patients. *Immunobiology* **204**, 517-523 (2001). <https://doi.org:10.1078/0171-2985-00060>

3 Downing, L. *et al.* A simple quantitative method for assessing pulmonary damage after x irradiation. *Radiat Res* **173**, 536-544 (2010). <https://doi.org:10.1667/RR1712.1>

4 Jeong, S. J. *et al.* The effect of sRAGE-Fc fusion protein attenuates inflammation and decreases mortality in a murine cecal ligation and puncture model. *Inflamm Res* **61**, 1211-1218 (2012). <https://doi.org:10.1007/s00011-012-0518-7>

5 Gachanja, N. N., Dorward, D. A., Rossi, A. G. & Lucas, C. D. Assays of Eosinophil Apoptosis and Phagocytic Uptake. *Methods Mol Biol* **2241**, 113-132 (2021). <https://doi.org:10.1007/978-1-0716-1095-4_10>
