# Supplementary figures and images for: Pharmacological inhibition of MutT homolog 1 (MTH1) in allergic airway inflammation as a novel treatment strategy
Source: Respir Res. 2025 Mar 14;26:101. doi: 10.1186/s12931-025-03175-z (PMC11909806; doi:10.1186/s12931-025-03175-z)

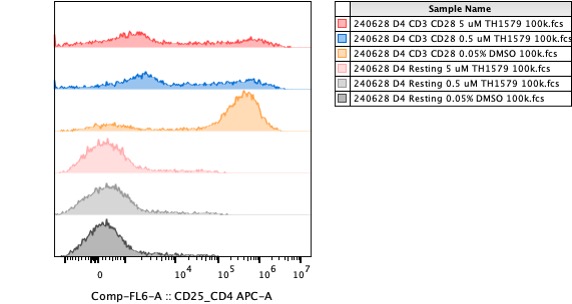

Supplement: Supplementary file 2 — Supplementary Material 2 [file 12931_2025_3175_MOESM2_ESM.jpg]

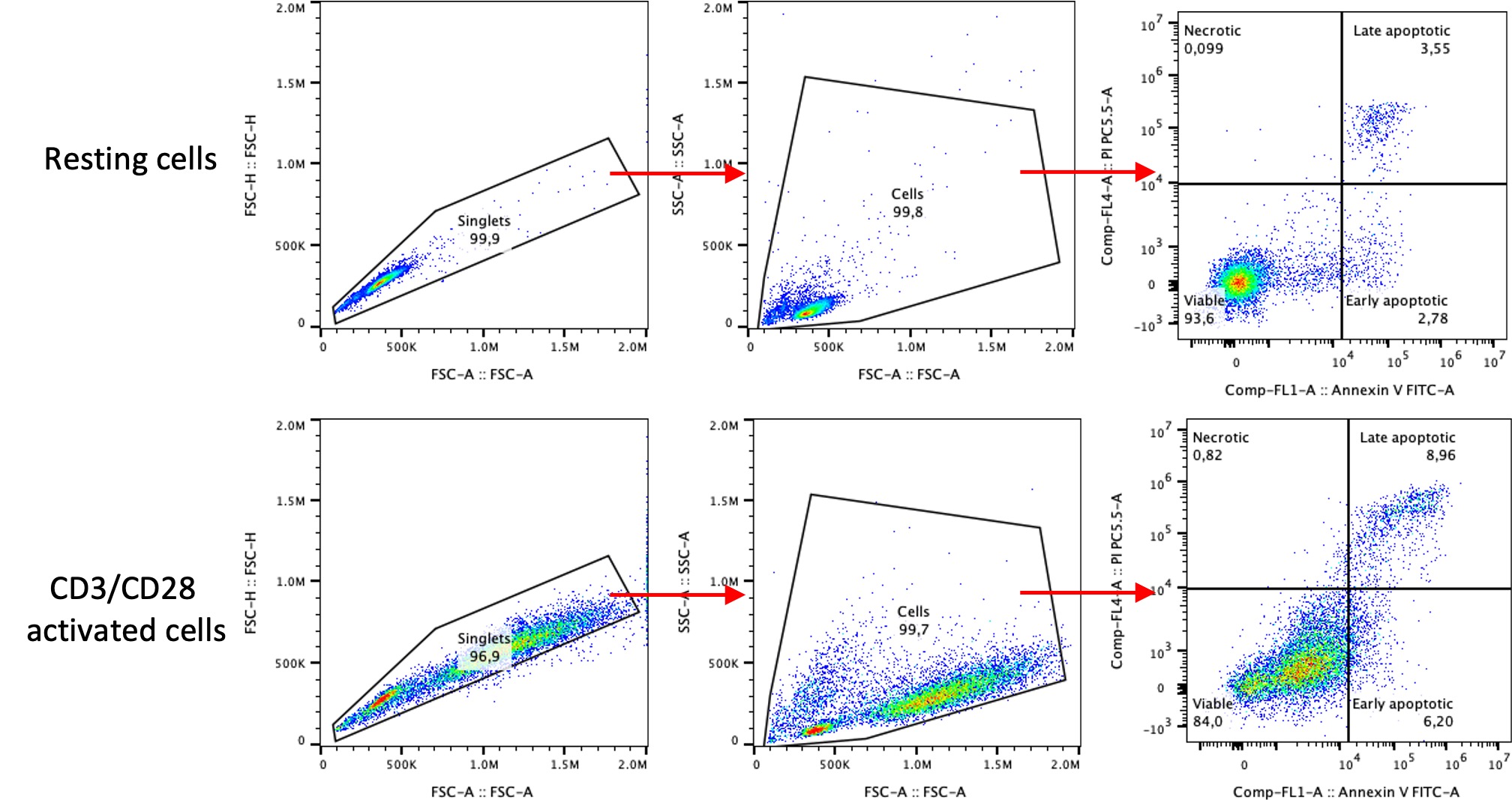

Supplement: Supplementary file 3 — Supplementary Material 3 [file 12931_2025_3175_MOESM3_ESM.jpg]

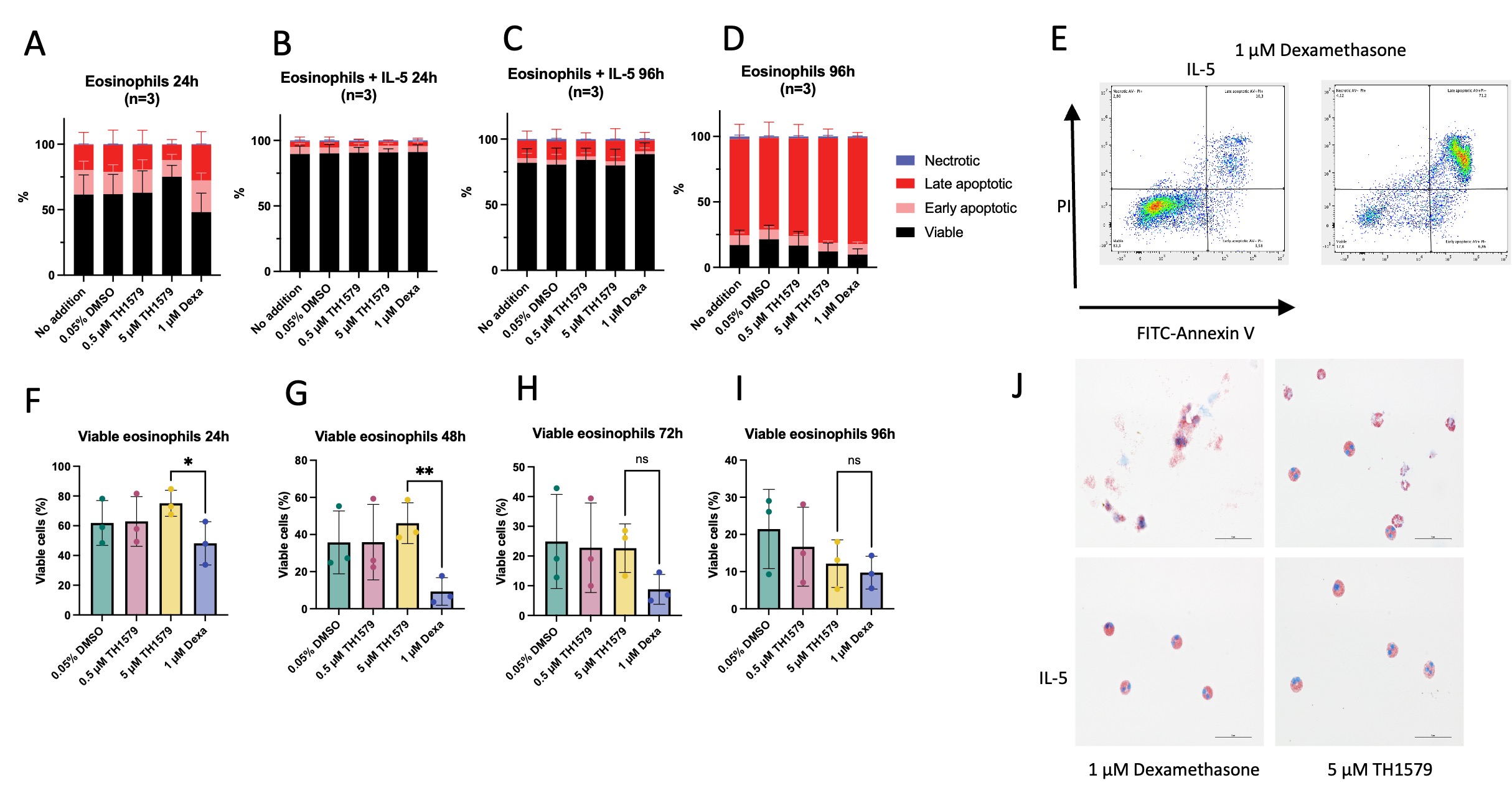

Supplement: Supplementary file 4 — Supplementary Material 4 [file 12931_2025_3175_MOESM4_ESM.jpg]

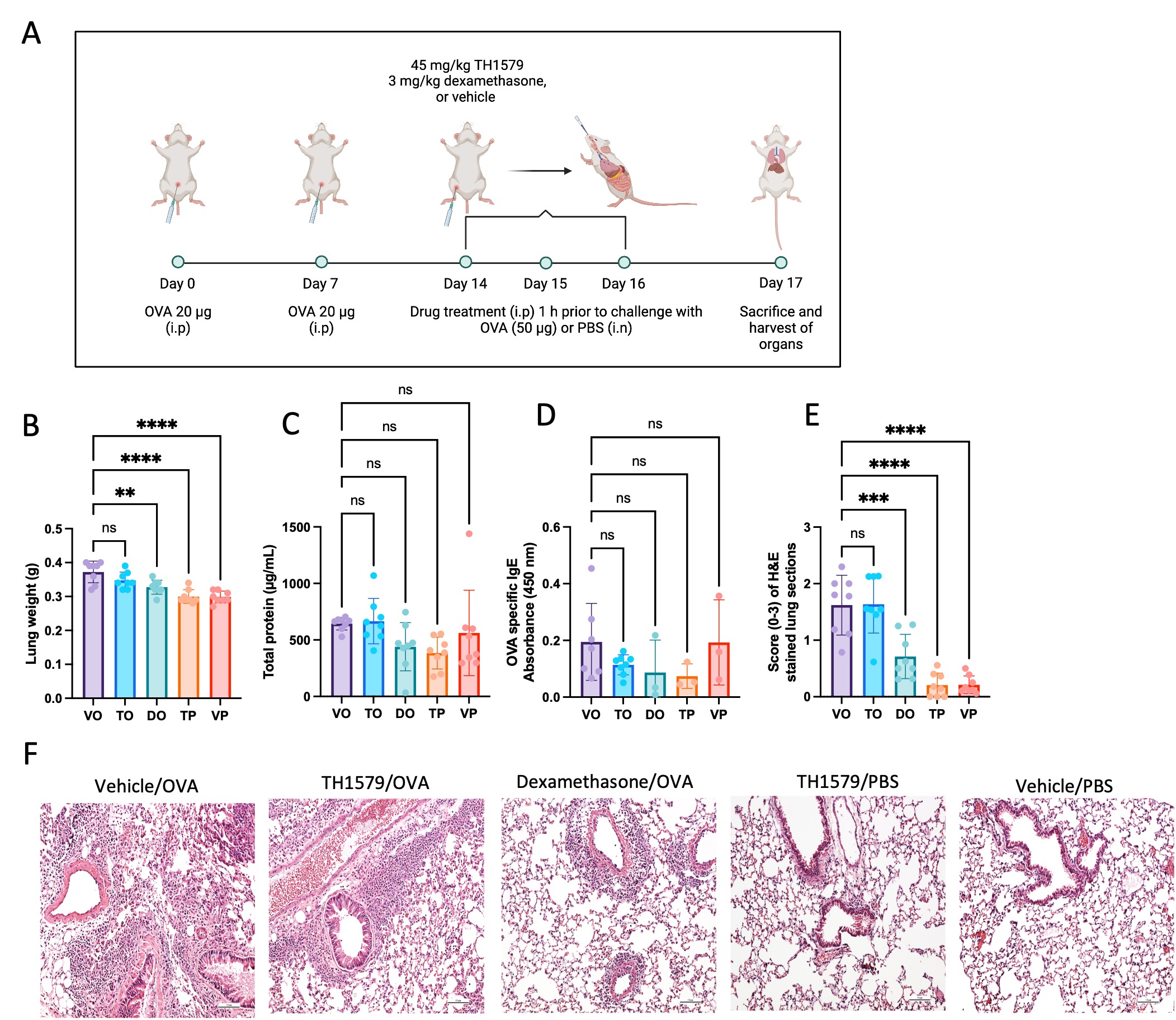

Supplement: Supplementary file 5 — Supplementary Material 5 [file 12931_2025_3175_MOESM5_ESM.jpg]

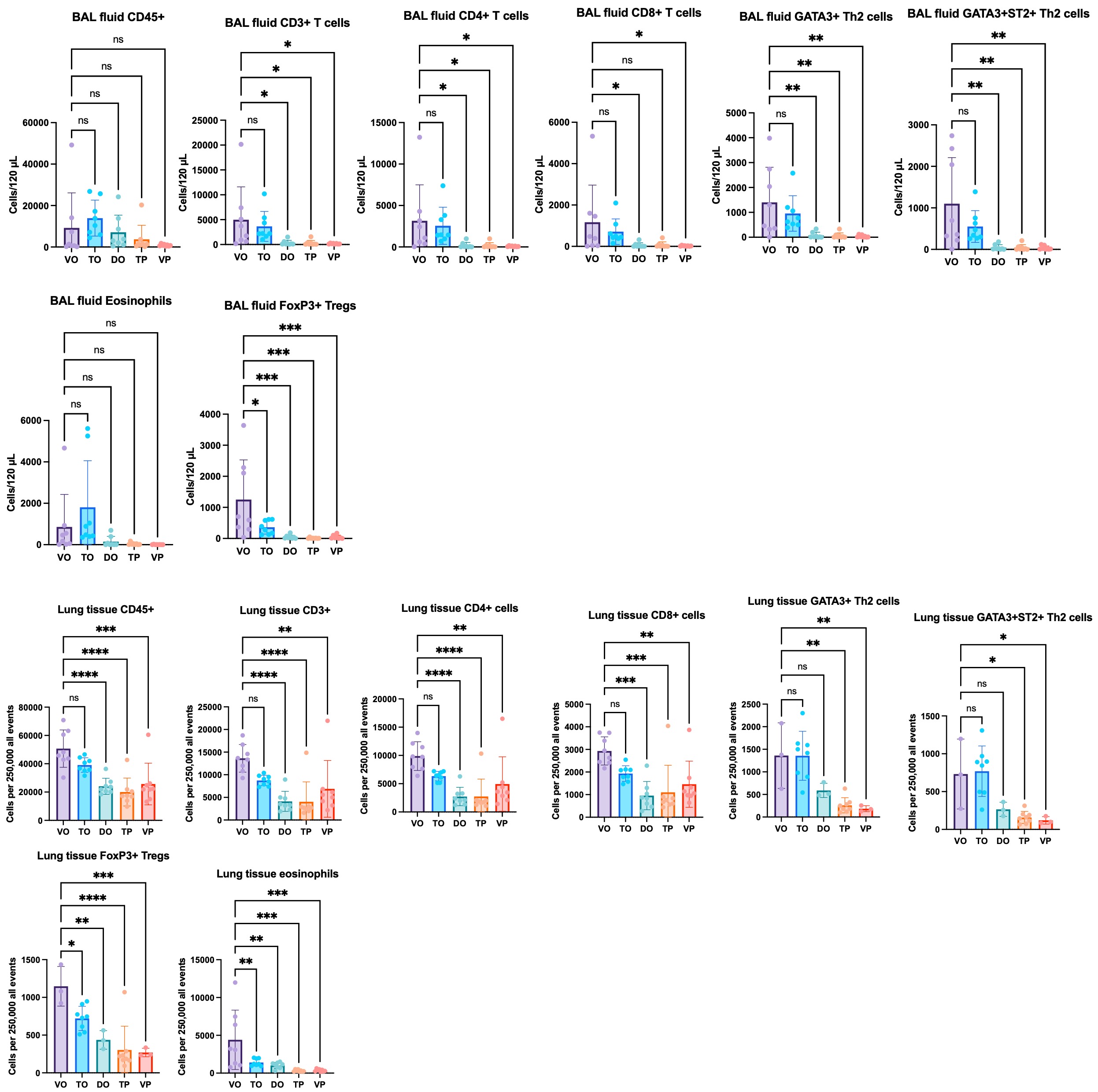

Supplement: Supplementary file 6 — Supplementary Material 6 [file 12931_2025_3175_MOESM6_ESM.jpg]

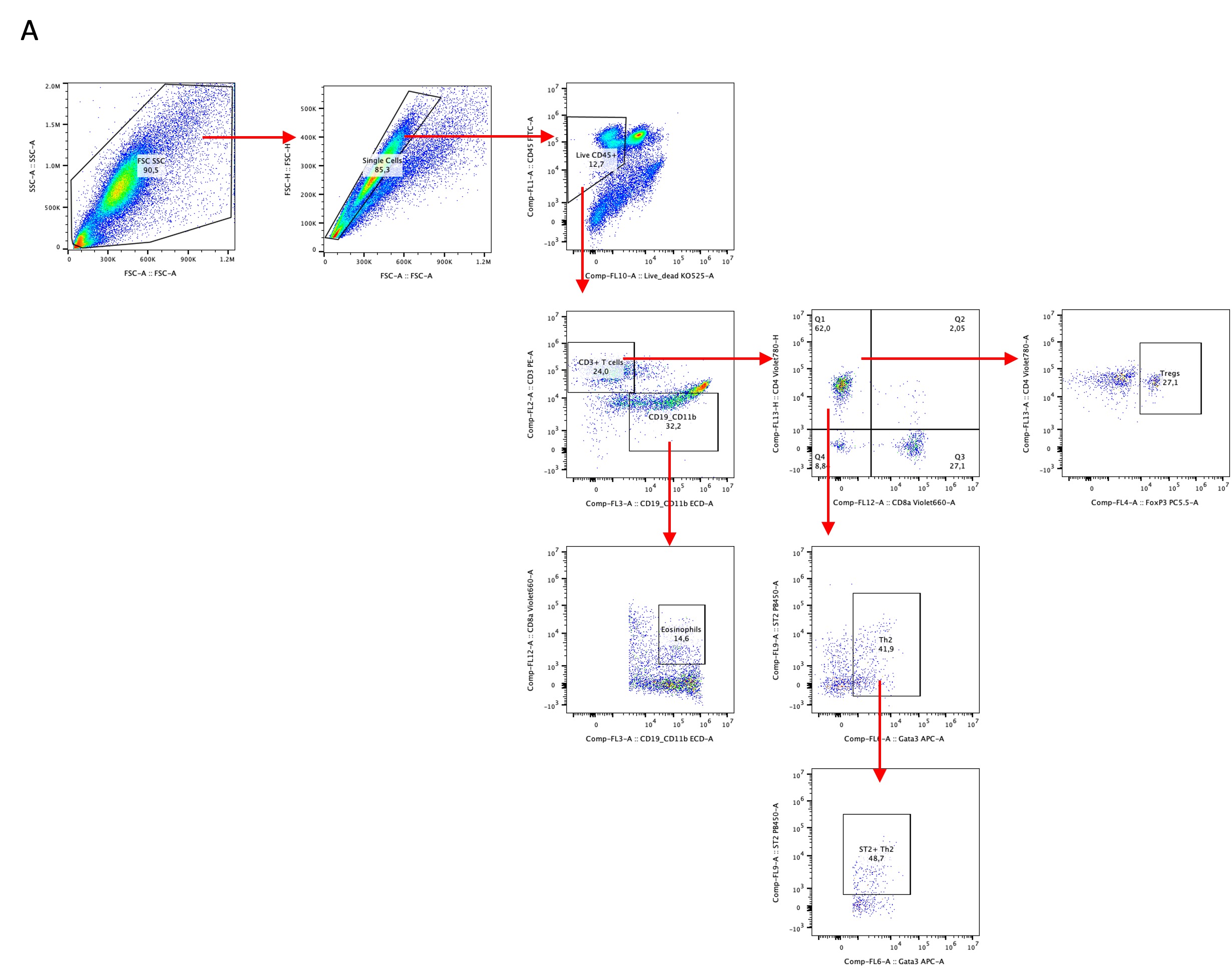

Supplement: Supplementary file 7 — Supplementary Material 7 [file 12931_2025_3175_MOESM7_ESM.jpg]

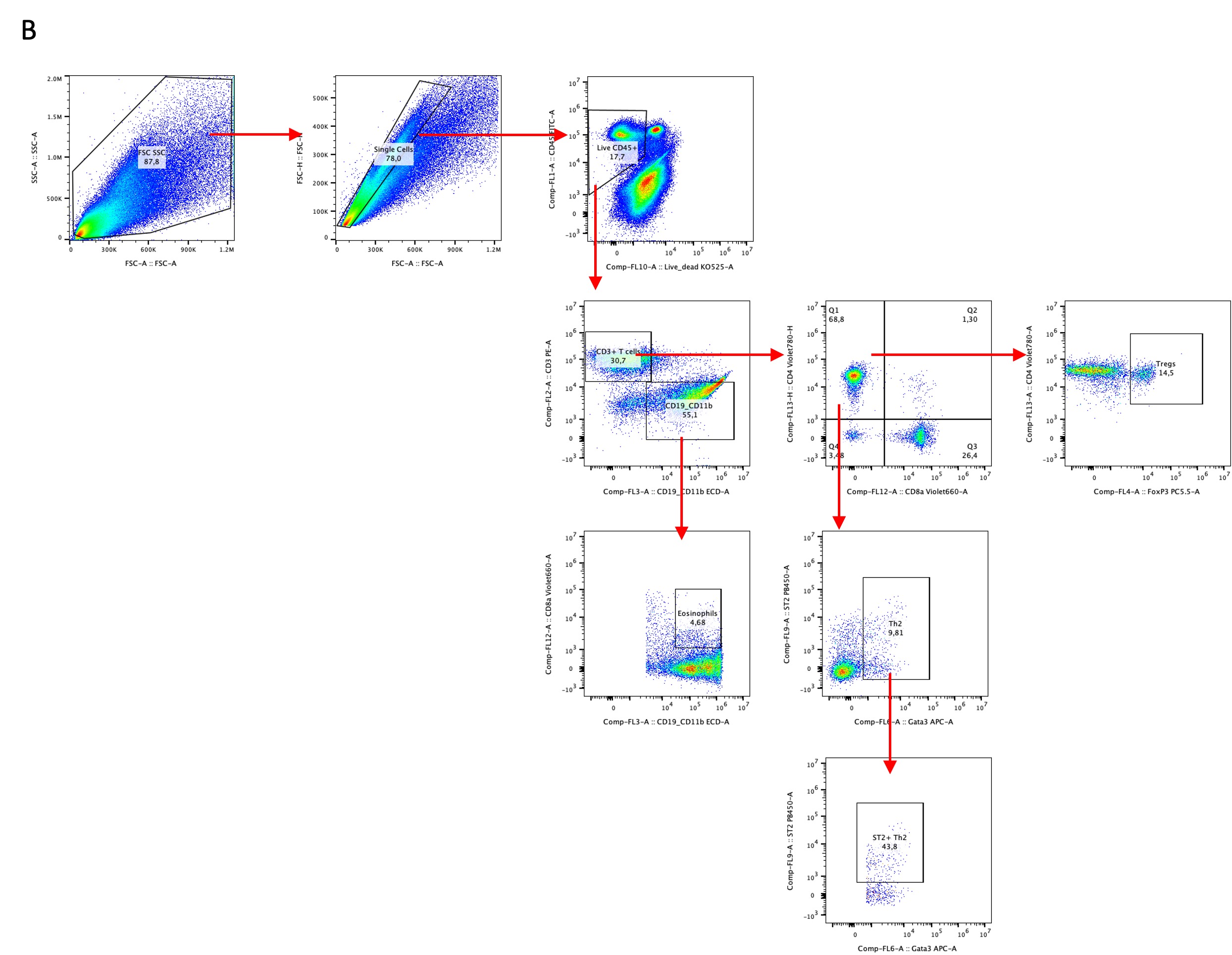

Supplement: Supplementary file 8 — Supplementary Material 8 [file 12931_2025_3175_MOESM8_ESM.jpg]

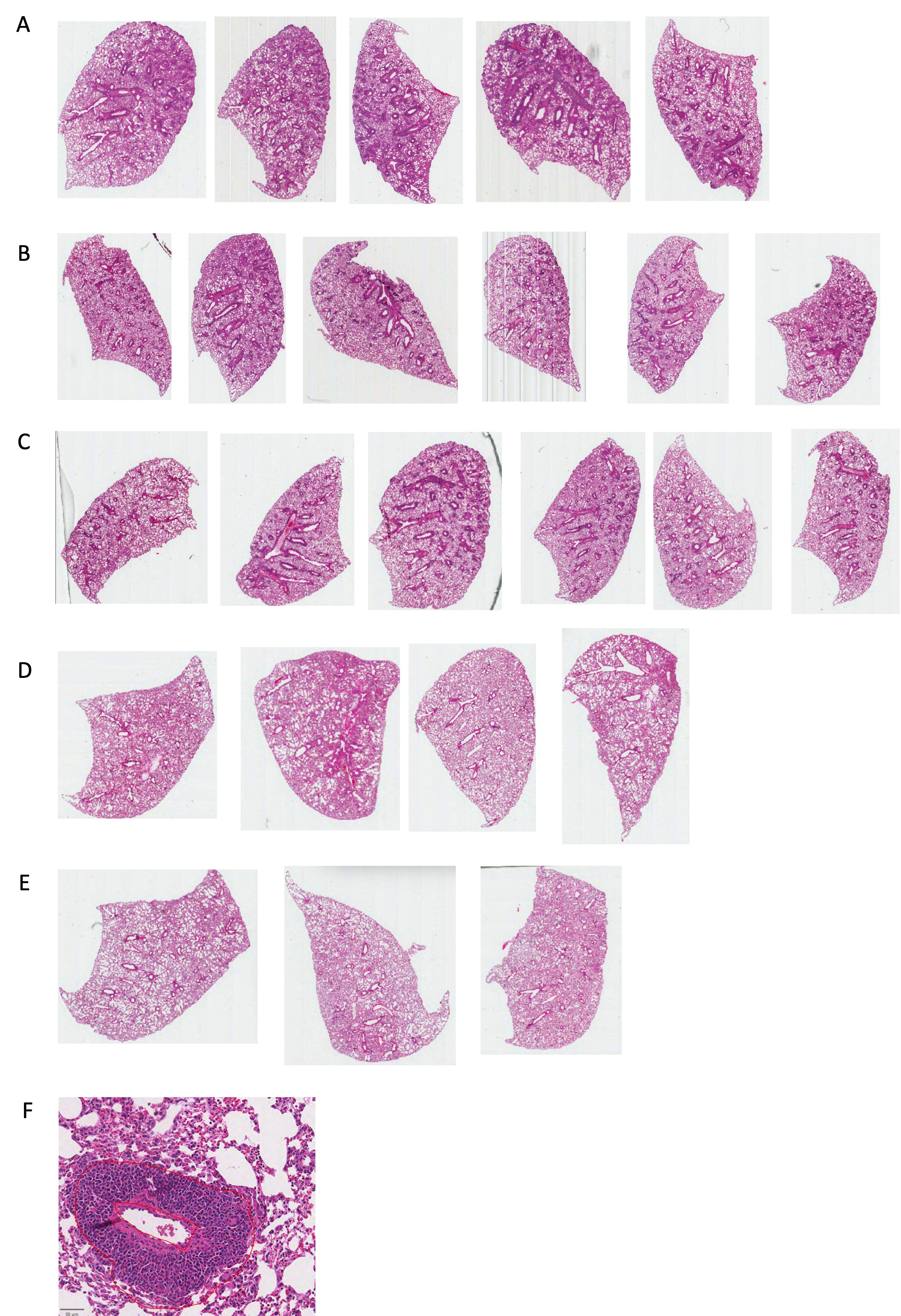

Supplement: Supplementary file 9 — Supplementary Material 9 [file 12931_2025_3175_MOESM9_ESM.jpg]

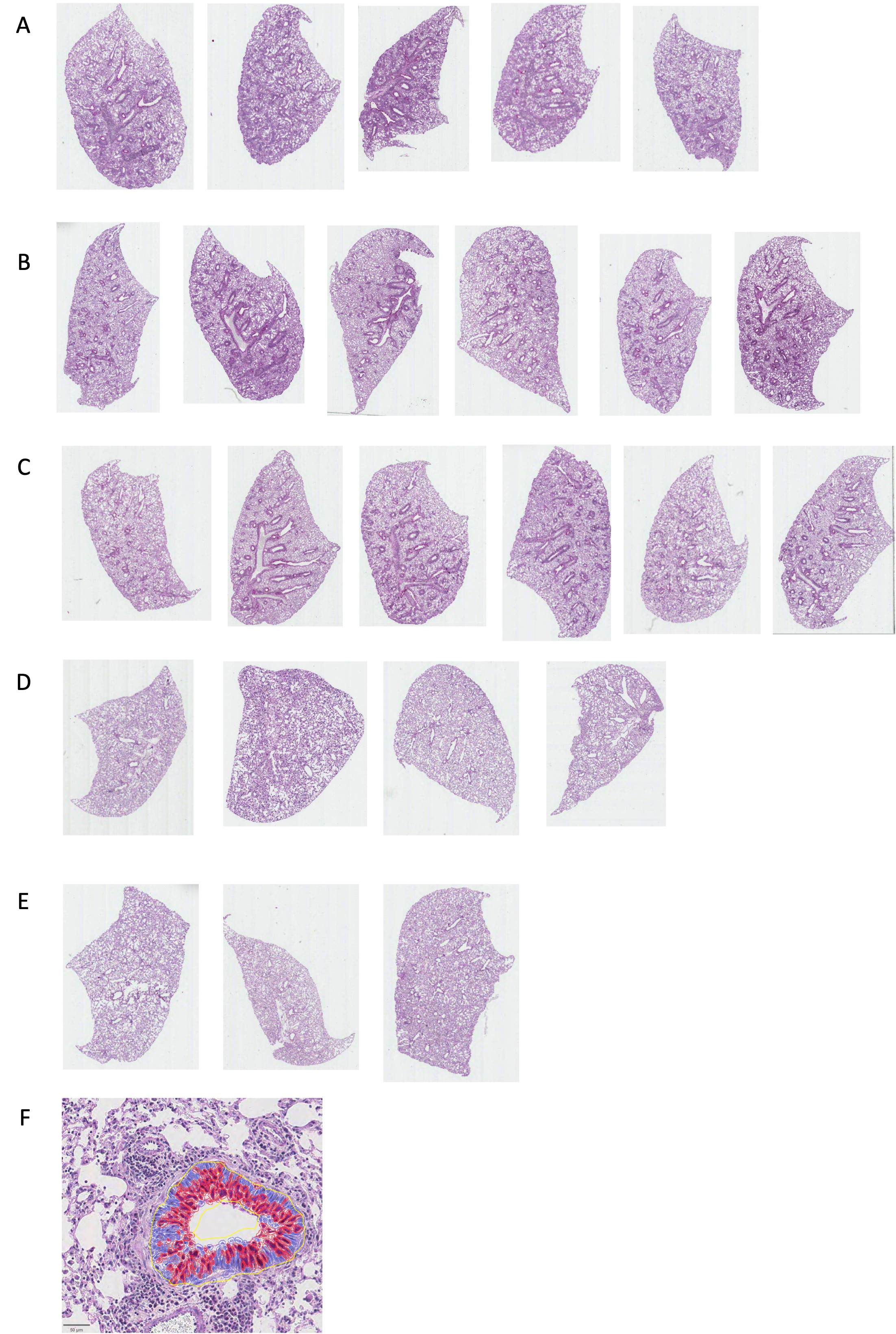

Supplement: Supplementary file 10 — Supplementary Material 10 [file 12931_2025_3175_MOESM10_ESM.jpg]

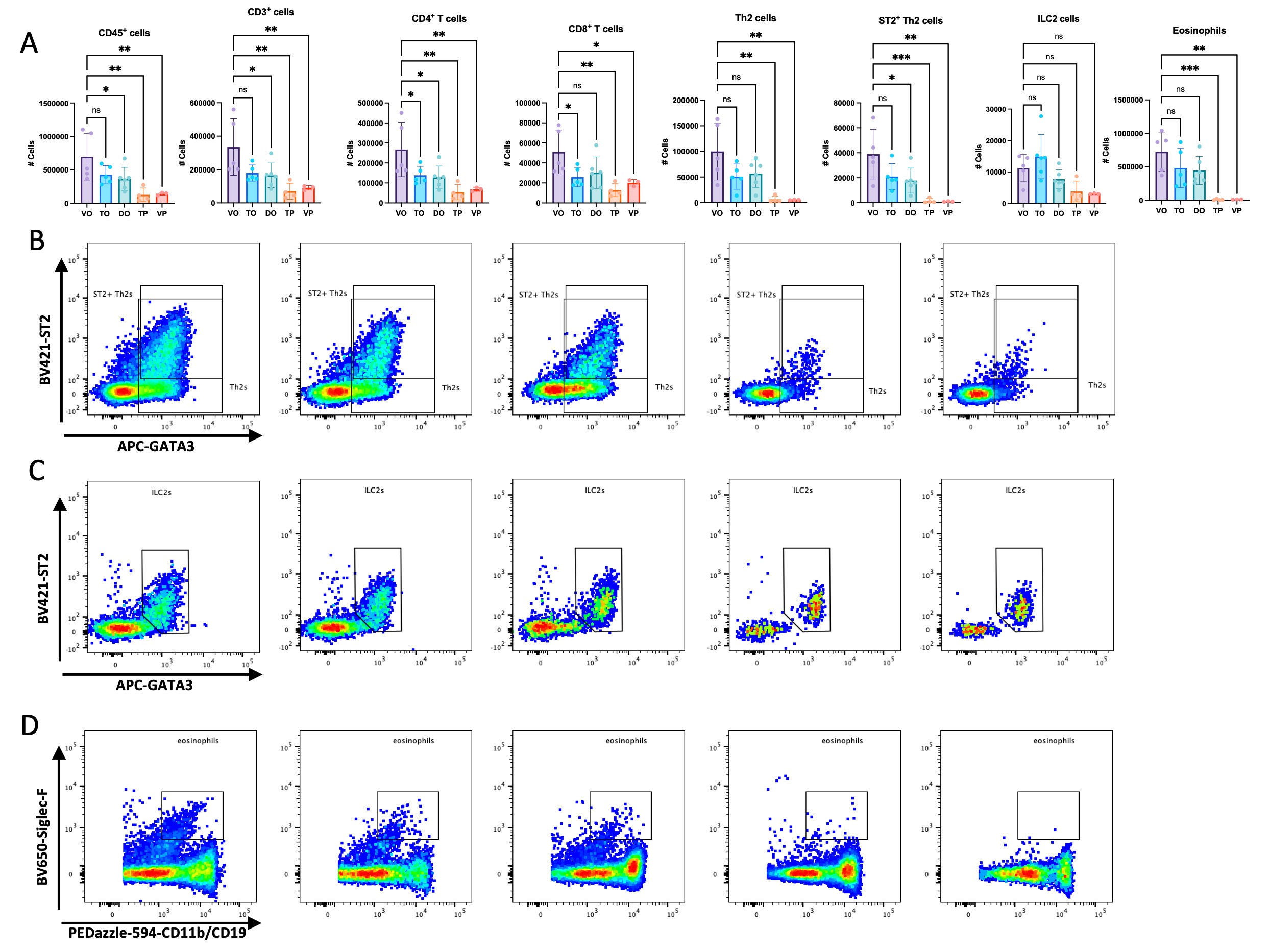

Supplement: Supplementary file 11 — Supplementary Material 11 [file 12931_2025_3175_MOESM11_ESM.jpg]

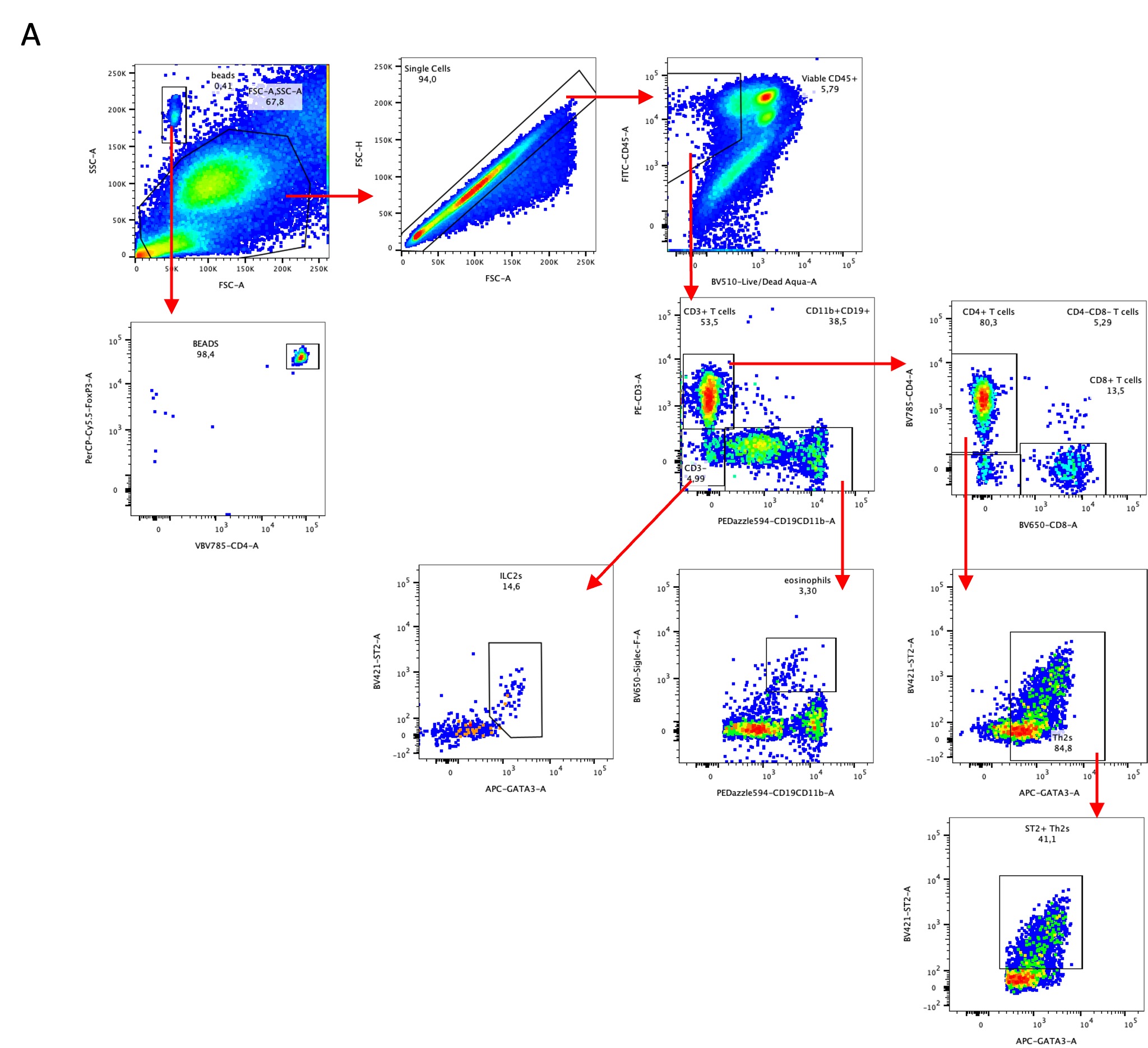

Supplement: Supplementary file 12 — Supplementary Material 12 [file 12931_2025_3175_MOESM12_ESM.jpg]

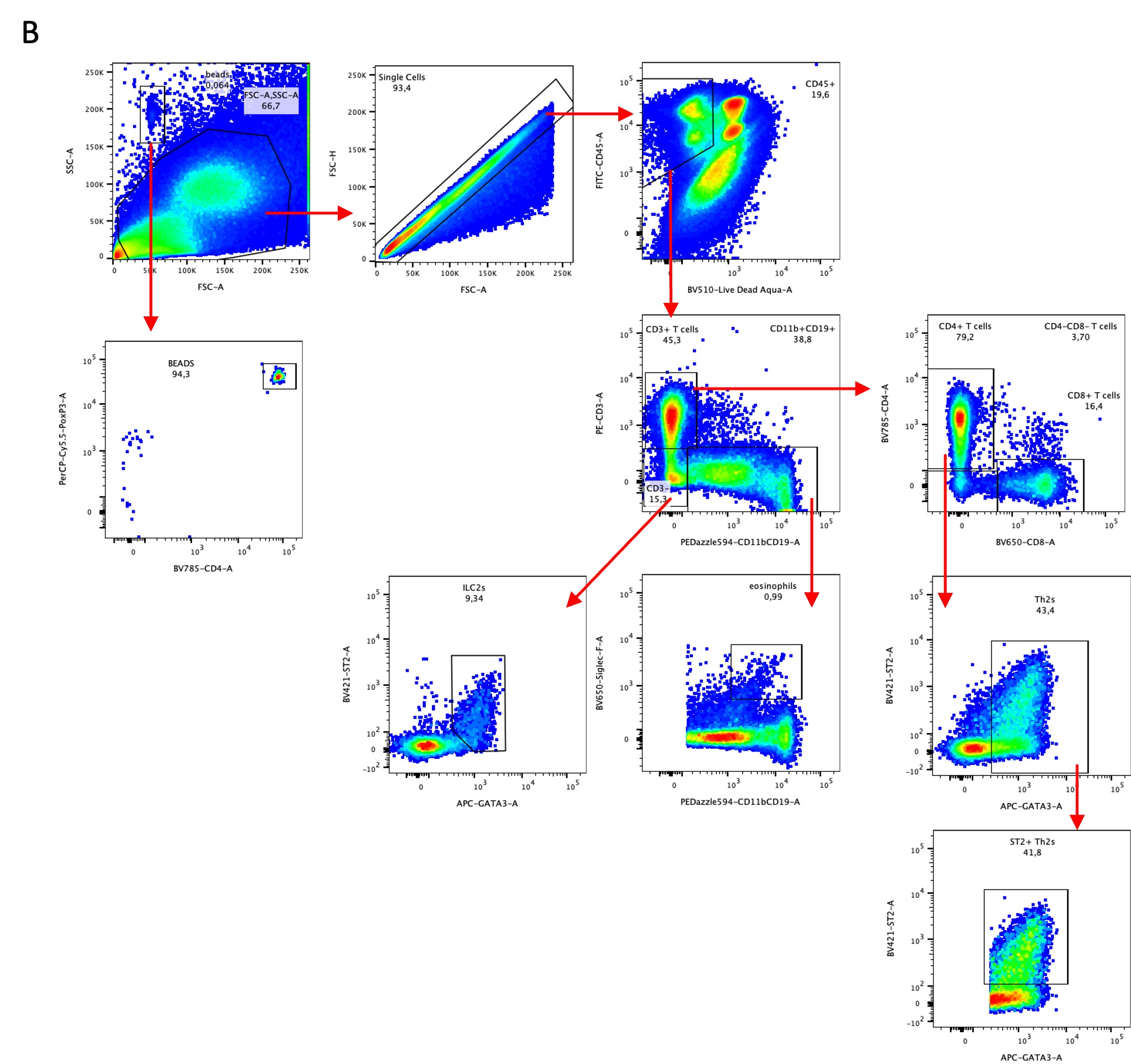

Supplement: Supplementary file 13 — Supplementary Material 13 [file 12931_2025_3175_MOESM13_ESM.jpg]

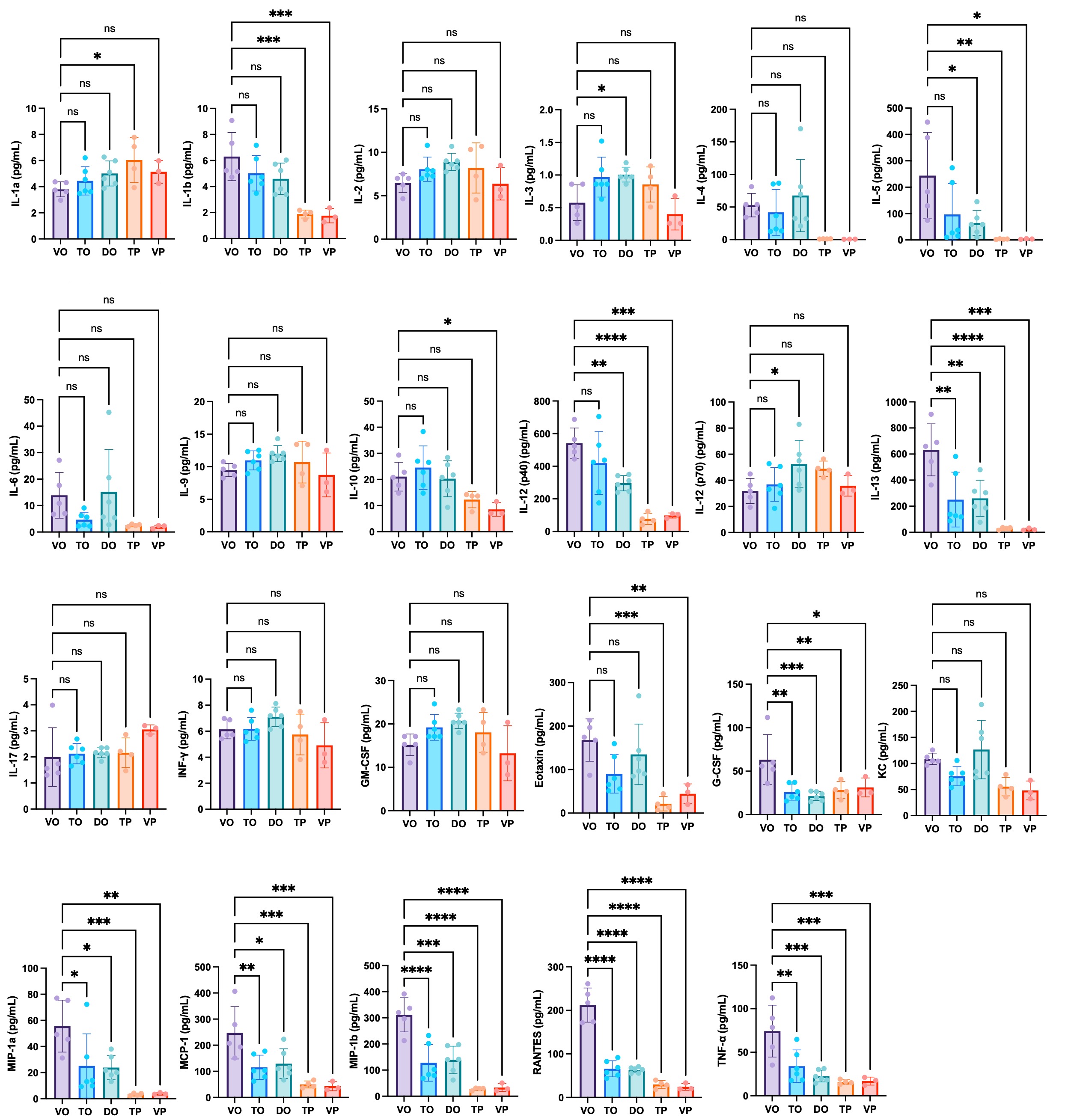

Supplement: Supplementary file 14 — Supplementary Material 14 [file 12931_2025_3175_MOESM14_ESM.jpg]

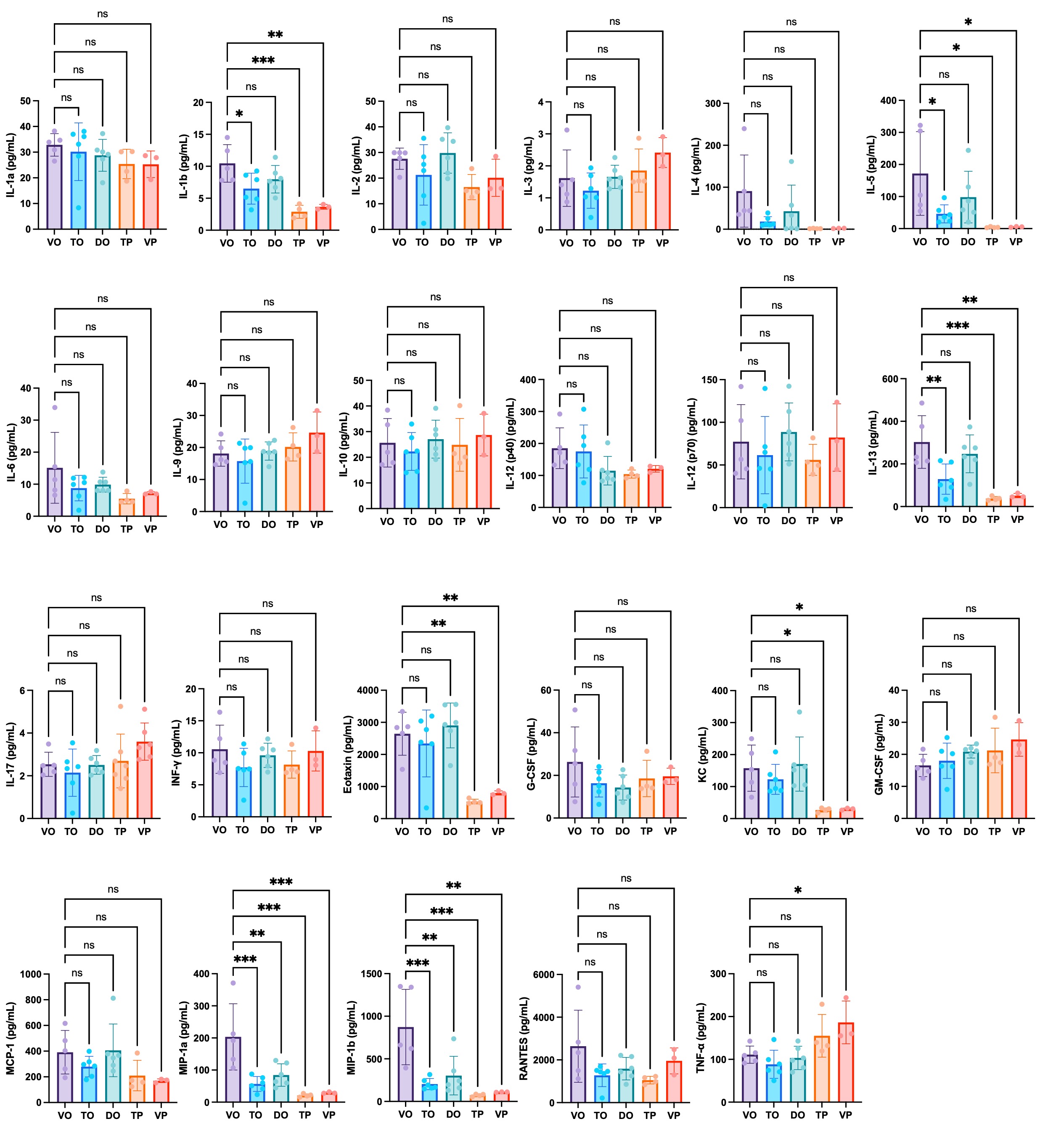

Supplement: Supplementary file 15 — Supplementary Material 15 [file 12931_2025_3175_MOESM15_ESM.jpg]

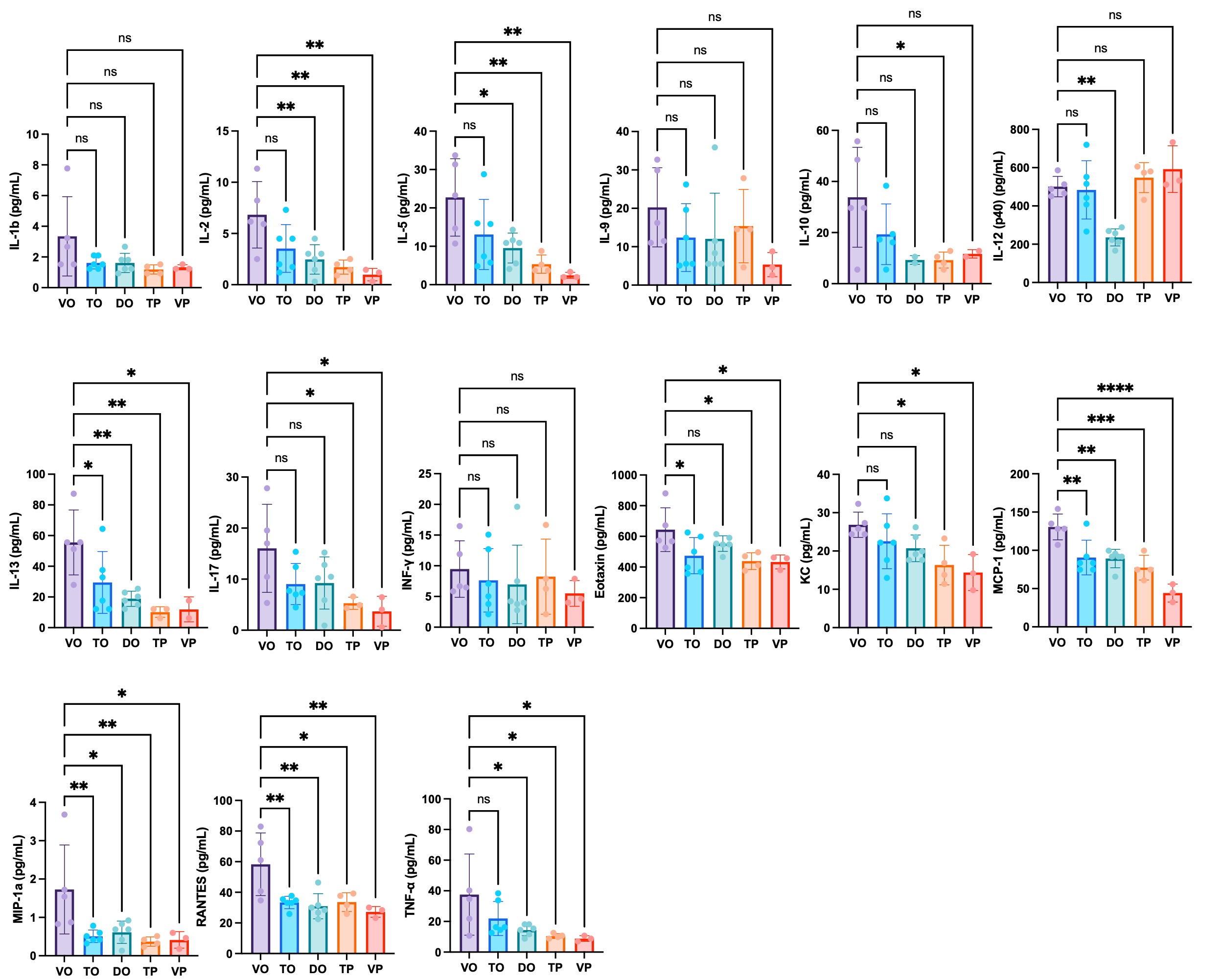

Supplement: Supplementary file 16 — Supplementary Material 16 [file 12931_2025_3175_MOESM16_ESM.jpg]

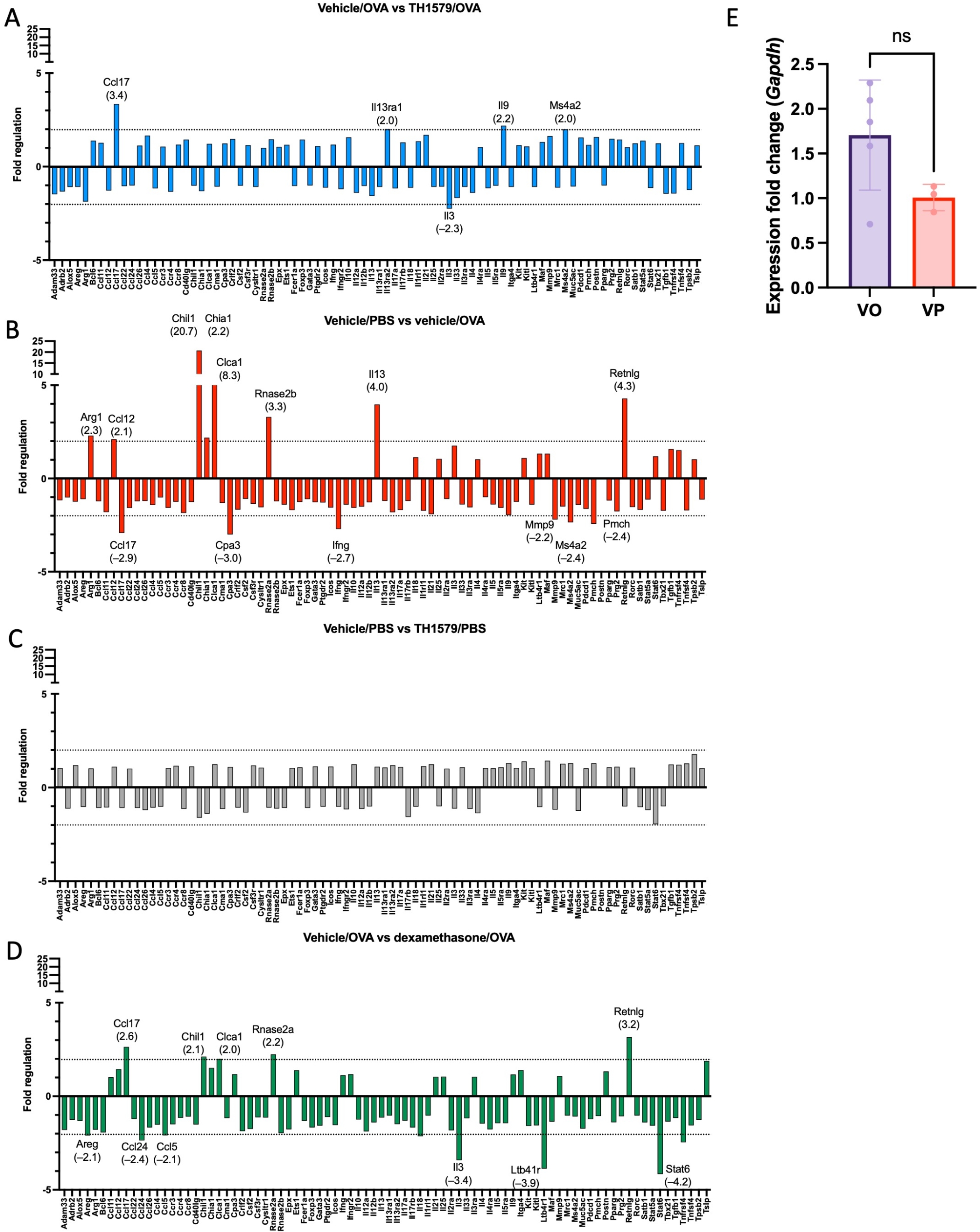

Supplement: Supplementary file 17 — Supplementary Material 17 [file 12931_2025_3175_MOESM17_ESM.jpg]
